# Is there an immunological cross-reactivity of antibodies to the myelin oligodendrocyte glycoprotein and coronaviruses?

**DOI:** 10.1093/braincomms/fcae106

**Published:** 2024-03-25

**Authors:** Kathrin Schanda, Sara Mariotto, Dagmar Rudzki, Angelika Bauer, Alessandro Dinoto, Patrizia Rossi, Sergio Ferrari, Sven Jarius, Brigitte Wildemann, Federica Boso, Bruno Giometto, Daniel Engels, Tania Kümpfel, Eva-Maria Wendel, Kevin Rostasy, Markus Reindl

**Affiliations:** Clinical Department of Neurology, Medical University of Innsbruck, 6020 Innsbruck, Austria; Neurology Unit, Department of Neurosciences, Biomedicine, and Movement Sciences, University of Verona, 37100 Verona, Italy; Clinical Department of Neurology, Medical University of Innsbruck, 6020 Innsbruck, Austria; Institute of Hygiene and Medical Microbiology, Medical University of Innsbruck, 6020 Innsbruck, Austria; Neurology Unit, Department of Neurosciences, Biomedicine, and Movement Sciences, University of Verona, 37100 Verona, Italy; Neurology Unit, St Bassiano Hospital, Bassano del Grappa, 36100 Vicenza, Italy; Neurology Unit, Department of Neurosciences, Biomedicine, and Movement Sciences, University of Verona, 37100 Verona, Italy; Molecular Neuroimmunology Group, Department of Neurology, University of Heidelberg, 69120 Heidelberg, Germany; Molecular Neuroimmunology Group, Department of Neurology, University of Heidelberg, 69120 Heidelberg, Germany; Neurology Unit, Trento Hospital, Azienda Provinciale per i Servizi Sanitari (APSS) di Trento, 38122 Trento, Italy; Neurology Unit, Trento Hospital, Azienda Provinciale per i Servizi Sanitari (APSS) di Trento, 38122 Trento, Italy; Institute of Clinical Neuroimmunology, University Hospital, Ludwig-Maximilians-Universität München, 81375 Munich, Germany; Institute of Clinical Neuroimmunology, University Hospital, Ludwig-Maximilians-Universität München, 81375 Munich, Germany; Department of Neuropediatrics, Olgahospital/Klinikum Stuttgart, 70174 Stuttgart, Germany; Paediatric Neurology, Witten/Herdecke University, Children's Hospital Datteln, 45711 Datteln, Germany; Clinical Department of Neurology, Medical University of Innsbruck, 6020 Innsbruck, Austria

**Keywords:** MOG, SARS-CoV-2, nucleocapsid protein, antibody, cross-reactivity

## Abstract

Recent reports indicated that myelin oligodendrocyte glycoprotein antibody-associated disease might be a rare complication after severe acute respiratory syndrome coronavirus 2 infection or vaccination. It is unclear whether this is an unspecific sequel of infection or vaccination or caused by possible immunological cross-reactivity of severe acute respiratory syndrome coronavirus 2 proteins and myelin oligodendrocyte glycoprotein. The aim of this study was therefore to elucidate whether there is an immunological cross-reactivity between severe acute respiratory syndrome coronavirus 2 spike or nucleocapsid proteins and myelin oligodendrocyte glycoprotein and to explore the relation of antibody responses against myelin oligodendrocyte glycoprotein and severe acute respiratory syndrome coronavirus 2 and other coronaviruses. We analysed serum samples from patients with severe acute respiratory syndrome coronavirus 2 infection and neurological symptoms with (myelin oligodendrocyte glycoprotein antibody-associated disease, *n* = 12) or without myelin oligodendrocyte glycoprotein-antibodies (*n* = 10); severe acute respiratory syndrome coronavirus 2 infection without neurological symptoms (*n* = 32); vaccinated patients with no history of severe acute respiratory syndrome coronavirus 2 infection and neurological symptoms with (myelin oligodendrocyte glycoprotein antibody-associated disease, *n* = 10) or without myelin oligodendrocyte glycoprotein-antibodies (*n* = 9); and severe acute respiratory syndrome coronavirus 2 negative/naïve unvaccinated patients with neurological symptoms with (myelin oligodendrocyte glycoprotein antibody-associated disease, *n* = 47) or without myelin oligodendrocyte glycoprotein-antibodies (*n* = 20). All samples were analysed for serum antibody responses to myelin oligodendrocyte glycoprotein, severe acute respiratory syndrome coronavirus 2, and other common coronaviruses (CoV-229E, CoV-HKU1, CoV-NL63 and CoV-OC43). Based on sample amount and antibody titres, 21 samples were selected for analysis of antibody cross-reactivity between myelin oligodendrocyte glycoprotein and severe acute respiratory syndrome coronavirus 2 spike and nucleocapsid proteins using affinity purification and pre-absorption. Whereas we found no association of immunoglobulin G and A myelin oligodendrocyte glycoprotein antibodies with coronavirus antibodies, infections with severe acute respiratory syndrome coronavirus 2 correlated with an increased immunoglobulin M myelin oligodendrocyte glycoprotein antibody response. Purified antibodies showed no cross-reactivity between severe acute respiratory syndrome coronavirus 2 spike protein and myelin oligodendrocyte glycoprotein. However, one sample of a patient with myelin oligodendrocyte glycoprotein antibody-associated disease following severe acute respiratory syndrome coronavirus 2 infection showed a clear immunoglobulin G antibody cross-reactivity to severe acute respiratory syndrome coronavirus 2 nucleocapsid protein and myelin oligodendrocyte glycoprotein. This patient was also seropositive for other coronaviruses and showed immunological cross-reactivity of severe acute respiratory syndrome coronavirus 2 and CoV-229E nucleocapsid proteins. Overall, our results indicate that an immunoglobulin G antibody cross-reactivity between myelin oligodendrocyte glycoprotein and severe acute respiratory syndrome coronavirus 2 proteins is rare. The presence of increased myelin oligodendrocyte glycoprotein-immunoglobulin M antibodies after severe acute respiratory syndrome coronavirus 2 infection may either be a consequence of a previous infection with other coronaviruses or arise as an unspecific sequel after viral infection. Furthermore, our data indicate that myelin oligodendrocyte glycoprotein-immunoglobulin A and particularly myelin oligodendrocyte glycoprotein-immunoglobulin M antibodies are a rather unspecific sequel of viral infections. Finally, our findings do not support a causative role of coronavirus infections for the presence of myelin oligodendrocyte glycoprotein-immunoglobulin G antibodies.

## Teaser

Schanda *et al*. report that an antibody cross-reactivity between myelin oligodendrocyte glycoprotein and severe acute respiratory syndrome coronavirus 2 proteins is rare. Their findings do not support a causative role of coronavirus infections for the presence of myelin oligodendrocyte glycoprotein antibodies.

## Graphical Abstract

**Figure:**
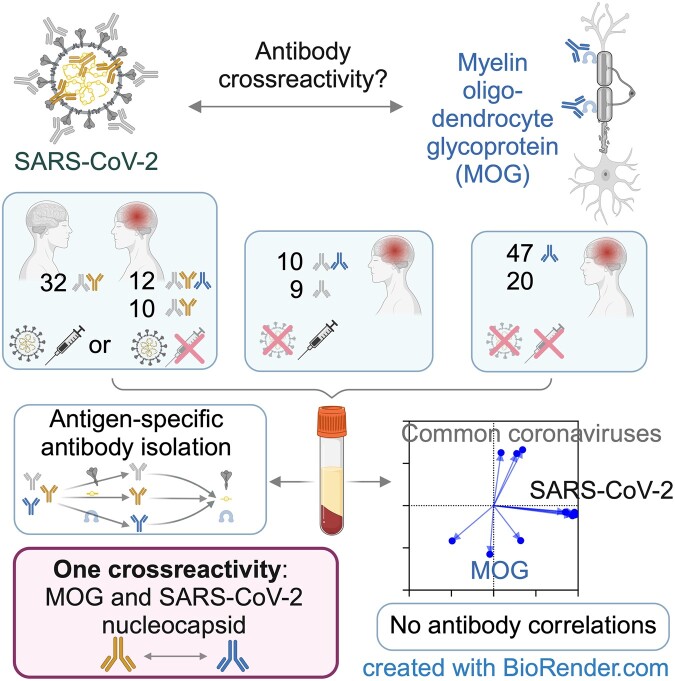

## Introduction

It is now well established that coronavirus disease 2019 (COVID-19) patients can present with neurological post-acute sequelae of COVID-19 (neuroPASC or also called Neuro-Covid), although severe acute respiratory syndrome coronavirus 2 (SARS-CoV-2) is rarely detected in the CSF or brain tissue.^1-3^ Neurological complications include stroke and immune-mediated disorders such as Guillain-Barré syndrome, autoimmune encephalitis, acute disseminated encephalomyelitis or myelitis.^3-5^ Therefore, it is likely that SARS-CoV-2, like many other viruses, can trigger post-infectious autoimmune diseases. Several recent publications reported patients with new occurring myelin oligodendrocyte glycoprotein IgG (MOG-IgG) associated disease (MOGAD), a rare recently defined neurological autoimmune diseases,^6^ after SARS-CoV-2 infection or vaccination indicating their possible role as a potential trigger of MOGAD.^7-22^ The association of MOGAD with infections or vaccination is well known and includes a broad range of infectious agents and vaccines.^23,24^

However, the potential mechanisms underlying these observations are still unclear. In this study, we have therefore analysed whether MOGAD is an unspecific sequel of SARS-CoV-2 infection/vaccination or caused by immunological cross-reactivity of MOG antibodies to SARS-CoV-2 spike (S) and/or nucleocapsid (N) proteins. We used immune-affinity-purified MOG and SARS-CoV-2 S and N specific antibodies from patients with MOGAD occurring after confirmed SARS-CoV-2 infection or vaccination and appropriate controls and analysed them for their immunological cross-reactivity. Furthermore, we investigated if pre-adsorption of serum samples with soluble SARS-CoV-2 S and N proteins reduced antibody binding to MOG and vice versa. To gain more insight into a possible role of coronavirus infections and MOGAD, we analysed the relation of antibody responses against MOG, SARS-CoV-2 and other common coronaviruses (CoV-229E, CoV-HKU1, CoV-OC43 and CoV-NL63) in a larger cohort of individuals with MOGAD and appropriate controls from before and during the pandemic.

## Materials and methods

### Samples and study participants

All serum samples were obtained from the participating centres (Neuropathology-Verona biobank, Verona, Italy; Molecular Neuroimmunology Group, Department of Neurology, University of Heidelberg, Heidelberg, Germany; Neurology Unit, Trento Hospital, Trento, Italy; Institute of Clinical Neuroimmunology, University Hospital, Ludwig-Maximillians-Universität München, Munich, Germany; Paediatric Neurology, Witten/Herdecke University, Children's Hospital Datteln, Datteln, Germany) and anonymized before sending them to Innsbruck, Austria. This study was approved by the local Bioethics Committee (Comitato Etico per la Sperimentazione Clinica, Azienda Ospedaliera Universitaria Integrata di Verona) (BIOB-NEU-DNA-2014, protocol 13582), the local review board of the University of Heidelberg, the local ethical committee (#A792, 13/10/2022) of University of Trento, the Institutional Review Board of the Ludwig-Maximillians-Universität Munich (protocol number 163-16 and 17-135) and the Ethics Committee of the Witten/Herdecke University (BIOMARKER-Study number AN4059). All patients or their caregivers gave written informed consent.

For this study, we used serum samples from patients with SARS-CoV-2 infection and neurological symptoms of the central nervous system (CNS) with (MOGAD, *n* = 12) or without MOG antibodies (CNS, *n* = 10); SARS-CoV-2 infection but no neurological symptoms (noCNS, *n* = 32); patients with SARS-CoV-2 vaccination without a history of SARS-CoV-2 infection and neurological symptoms with (MOGAD, *n* = 10) or without MOG antibodies (CNS, *n* = 9); and SARS-CoV-2 negative/naïve unvaccinated patients with neurological symptoms with (MOGAD, *n* = 47) or without MOG antibodies (CNS, *n* = 20). MOGAD was diagnosed according to recently published diagnostic guidelines by the respective centres.^6,25^ The median time from neurological event to sampling was 0 (range 0–41) months and the majority of samples (71%) were obtained within 3 months after neurological event. Overall, 21 (15%) of all patients received immunosuppressive treatments at the time of sampling (19 steroids, one azathioprine, one rituximab). The median number of freeze-thaw cycles was 2 (range 1–5), and samples were stored in dedicated aliquots for each analysis to avoid further freeze-thaw cycles. Clinical and serological data including the year of sampling of all patients are shown in Table 1. A detailed clinical description of a subset of these patients was recently published.^13,17,22,26^

**Table 1:** Clinical and serological data of patient samples analysed for the relation of antibody levels against MOG, SARS-CoV-2 and other common coronaviruses

	SARS-CoV-2 infection			SARS-CoV-2 vaccination		SARS-CoV-2 negative	
	No CNS	CNS	MOGAD	CNS	MOGAD	CNS	MOGAD
Number	32	10	12	9	10	20	47
Females	10 (31%)	3 (30%)	7 (58%)	4 (44%)	2 (20%)	13 (65%)	21 (45%)
Age (years)^a^	66 (34–90)	49 (9–72)	28 (7–85)	38 (15–71)	28 (2–67)	12 (0–58)	9 (1–62)
Children	0 (0%)	4 (40%)	4 (33%)	1 (11%)	4 (40%)	16 (80%)	31 (66%)
CNS symptoms							
ADEM	0	0	3	1	1	3	15
ON	0	0	4	2	4	5	7
Myelitis	0	1	1	1	1	1	9
ON + myelitis	0	0	1	0	1	0	4
BS/Cerebellar	0	1	1	0	1	1	3
Encephalitis	0	3	2	1	2	2	9
NMOSD	0	0	0	1	0	4	0
MS	0	1	0	2	0	3	0
OND	0	4	0	1	0	1	0
noCNS	32	0	0	0	0	0	0
Time since event^c^	n.a.	1 (0–12)	1 (0–13)	4 (0–8)	0 (0–3)	0 (0–21)	1 (0–41)
Immunotherapy	0 (0%)	0 (0%)	4 (33%)	2 (22%)	3 (30%)	3 (15%)	9 (19%)
Freeze-thaw cycle	3 (2–5)	1 (1–2)	1 (1–5)	1 (1–5)	2 (1–5)	2 (1–5)	2 (1–5)
MOG-IgG							
Negative	28 (88%)	10 (100%)	0 (0%)	9 (100%)	0 (0%)	20 (100%)	0 (0%)
Low positive	4 (12%)	0 (0%)	6 (50%)	0 (0%)	4 (40%)	0 (0%)	14 (30%)
Clear positive	0 (0%)	0 (0%)	6 (50%)	0 (0%)	6 (60%)	0 (0%)	33 (70%)
Acute infection							
SARS-CoV-2	32	10^b^	12^b^	0	0	0	0
Other	0	0	0	0	1	1^b^	17^b^
Sample from:							
Before 2020	0	0	0	0	0	10	34
2020	32	0	0	0	0	4	4
2021	0	3	5	3	7	3	3
2022/2023	0	7	7	6	3	0	4
SARS-CoV-2 N-Ig	28 (88%)	6 (60%)	12 (100%)	0 (0%)	0 (0%)	1 (5%)	0 (0%)
SARS-CoV-2 S-Ig	26 (81%)	10 (100%)	11 (92%)	8 (89%)	9 (90%)	0 (0%)	0 (0%)
CoV-229E-Ig	26 (81%)	6 (60%)	9 (75%)	9 (100%)	7 (70%)	16 (80%)	33 (70%)
CoV-HKU1-Ig	30 (94%)	7 (70%)	10 (83%)	8 (89%)	7 (70%)	16 (80%)	27 (57%)
CoV-OC43-Ig	22 (69%)	3 (30%)	7 (58%)	8 (89%)	5 (50%)	12 (60%)	31 (66%)
CoV-NL63-Ig	27 (84%)	9 (90%)	11 (92%)	8 (89%)	9 (90%)	17 (85%)	39 (83%)
Samples tested for immunological cross-reactivity:							
Number	0	0	11	2	4	0	4

ADEM, acute disseminated encephalomyelitis; BS, brainstem, CNS, neurological diseases; CoV, coronavirus; Ig, immunoglobulin; IgG, immunoglobulin G; MOG, myelin oligodendrocyte glycoprotein; MOGAD, MOG antibody-associated disease; MS, multiple sclerosis; N, SARS-CoV-2 nucleocapsid protein; NMOSD, neuromyelitis optical spectrum disorder; n.a., not applicable; noCNS, no neurological symptoms; ON, optic neuritis; OND, other neurological diseases; SARS-CoV-2, severe acute respiratory syndrome coronavirus; S, SARS-CoV-2 spike protein.

^a^Median (range).

^b^Sample taken at first neurological presentation.

^c^Time since neurological event (months).

Thereof, 21 samples were analysed for antibody cross-reactivity between MOG and SARS-CoV-2 S and N proteins using affinity purification and pre-absorption (Table 1 and Supplementary Table 1). Furthermore, follow-up samples of 17 MOGAD patients (7 with COVID-19 and 10 with other infections) were also included to compare the temporal pattern of antibody responses.

### Immunoassays

Live cell-based assays (CBA) for MOG antibodies were performed according to published protocols.^27,28^ HEK293 cells were transiently transfected with the following plasmids: pEGFP-N1-MOGα1 (expression: native MOGα1 isoform),^29^ pcDNA3.1(+)-SARS-COV-2 S (Invitrogen, expression: SARS-CoV-2 S protein wildtype), pcDNA3.1 SARS-CoV-2 N (expression: SARS-CoV-2 N protein wildtype; gift from Jeremy Luban, Addgene plasmid # 158079; http://n2t.net/addgene:158079; RRID:Addgene_158079)^29,30^ and as depletion control plasmid pEGFP-N1 (expression: enhanced green fluorescent protein) and an empty pcDNA3.1 control plasmid for live CBA.

Live CBAs were performed 24 h post-transfection according to previously published protocols, using a starting dilution of 1:20 for sera, pure pre-diluted (1:20) serum supernatants from depletion experiments and undiluted purified antibody fractions.^28^ For fixed CBAs (SARS-CoV-2 N), cells were fixed with 100% methanol (−20°C) for 10 min at room temperature (RT), followed by three washes with phosphate buffered saline (PBS) and 1 h of blocking (5% normal goat serum, 1% bovine serum albumin in PBS).

Supernatants (final dilution: 1:200) and isolated fractions (final dilution: 1:2) were diluted in dilution buffer (1% normal goat serum–1% bovine serum albumin in PBS) and added overnight (4°C). After three washes with PBS, secondary antibody was added in dilution buffer for 30 min at RT, removed and the plate washed with PBS before analysis.

For the detection of bound human antibodies in both live and fixed CBA, we used anti-human IgG Fc-specific (Alexa 594, Jackson Laboratories 109-586-098, 1:750), anti-human IgA (Alexa 594, Jackson Laboratories 109-586-011, 1:750) and anti-human IgMµ-specific (Alexa 594, Jackson Laboratories 109-585-129, 1:750). Analysis of CBAs was performed visually with a fluorescence microscope (Leica DMI4000B), resulting in semi-quantitative titre values for live CBA. Images were captured at identical exposure times for both live CBAs (1.4 s) and lower exposure times for fixed CBA (570 ms, software Application Suite 4.13 by Leica). The live CBA for antibodies (IgG, IgM and IgA) against SARS-CoV-2 S protein showed a high correlation with commercial antibody assays for SARS-CoV-2 S protein (Supplementary Fig. 1).

Serum IgG antibodies against SARS-CoV-2 S1 and N proteins (both wildtype, original Wuhan variant) were determined by commercial ELISA kits [Euroimmun, Lübeck, Germany, Catalogue # EI 2606-9601–10 G Anti-SARS-CoV-2-QuantiVac-ELISA (IgG) and EI 2606-9601-2 G Anti-SARS-CoV-2-NCP-ELISA (IgG)]. Both assays were performed according to the manufacturer’s instructions, using serum and depleted/control serum supernatants at a final dilution of 1:100 and undiluted isolated antibodies. Preliminary experiments using positive sera diluted in 100% neutralized elution buffer showed no loss of reactivity compared to the dilution buffer included in the commercial kits. Analysis of 10% ultra-low IgG foetal calf serum (FCS) in PBS showed no unspecific reactivity in both ELISA kits.

Additionally, antibody analysis of selected sera and/or purified IgG fractions were performed against the following antigens: SARS-CoV-2-N (Invitrogen RP87665), CoV-229E (HCoV-229E NP, Sino Biologicals 40640), CoV-OC43 (HCoV-OC43 NP, Sino Biologicals 40643), CoV-HKU1 (HCoV-HKU1 NP, Sino Biologicals 40642) and overlapping 20mer peptides of SARS-CoV-2 N 1-242 (synthetized by Genscript, Uniprot P0DTC9). The ELISA protocol was based on published protocols^31^ with the modifications of 5 µg/ml in PBS of the appropriate antigen for coating and 37°C incubation temperature without shaking for the incubation of sera (1:100) or undiluted purified Ig fractions and secondary antibody (anti-human IgG horse radish peroxidass conjugated, 1:6000, Cytiva).

Serum total Ig antibodies against SARS-CoV-2 [RBD, S1 and S(trimer)] and N proteins and other coronaviruses (CoV-229E, CoV-HKU1, CoV-OC43 and CoV-NL63) were measured using the Coronavirus Ig Total Human 11-Plex ProcartaPlex™ Panel (Invitrogen, EPX110-16000-901) according to the manufacturer’s instructions. Samples stored at −80°C were thawed on ice and then used immediately. Fifty microlitres of magnetic beads per well were washed with 150 µl 1 × wash buffer using a hand-held magnetic plate washer. Then, 25 µl of assay diluent (each well), 25 µl of standards or 25 µl of pre-diluted (1:1000) serum samples were added to the according wells and incubated for 2 h at RT on a shaker at 600 rounds per minute (rpm). After washing twice, 25 µl of a detection antibody mixture was added and incubated for 30 min at RT on a shaker. After two washes, 120 µl reading buffer was added into each well and after a 5-min incubation period whilst shaking at RT, fluorescence intensity was measured on a Luminex MAGPIX instrument (Software: xPonent 4.2 and ProcartaPlex Analyst 1.0). In samples where analytes were not detectable or their concentrations exceeded the highest standard, the values of the lowest or highest standards were used for statistical analyses. Antibody responses against SARS-CoV-2 N and S proteins were scored as negative or positive using standards provided in the assay kit according to the manufacturer’s recommendations. Because no reference values were provided by the manufacturer for antibodies against other coronaviruses (CoV-229E, CoV-HKU1, CoV-OC43 and CoV-NL63), we used the lower 95% confidence interval of the geometric mean antibody titres of the SARS-CoV-2-negative population as recommended by other researchers.^32^ Consequently, the positivity cut-offs were as follows: 3400 U/ml for CoV-229E, 3400 U/ml for CoV-HKU1, 3600 U/ml for CoV-OC43 and 3800 U/ml for CoV-NL63. As can be seen in Supplementary Fig. 2, the majority of individuals were seropositive for these antibodies after an age of 10 years.

### Affinity purification of human antibodies against MOG and SARS-CoV-2 S and N

Affinity purification of antigen-specific human antibodies from sera was performed using either live transfected cells expressing native surface antigens (MOG and SARS-CoV-2 S) or recombinant proteins (SARS-CoV-2 N, Invitrogen RP-87665) and in-house produced canine distemper virus N protein^33^ bound to M-280-tosylactivated dynabeads (Invitrogen 14203). Briefly, diluted sera were incubated with antigen expressing cells/dynabeads coupled to antigens or controls. After removal of antigen bearing cells/dynabeads, diluted serum supernatants with or without (control) depleted antibodies were stored at −20°C until analysis with CBA and ELISA. After several washing steps, antigen-specific or control affinity-purified antibodies were eluted from cells/dynabeads and stored at −20°C, either undiluted for ELISA analysis or with the addition of ultra-low IgG FCS (Gibco 16250-078) to a concentration of 10% for CBA.

For antibody isolation using transfected cells, serum samples were diluted in 10% ultra-low IgG FCS in PBS (2 ml) while isolation with beads was performed with serum samples diluted in 0.1% bovine serum albumin (Sigma) in PBS (1:20). For affinity purification of antibodies against MOG and SARS-CoV-2 S proteins, HEK293 cells were transiently transfected with the plasmids encoding MOG, SARS-CoV-2 S and enhanced green fluorescent protein (control), respectively. Twenty-four hours post-transfection, cells were harvested, brought to a concentration of 6 × 10^6^ ml in 10% ultra-low IgG FCS in PBS and placed on an overhead rotator at RT for 30 min recovery. After recovery, 12 × 10^6^ cells of each transfection (MOG, SARS-CoV-2 S, and enhanced green fluorescent protein) were placed into a separate tube and spun down at 500 g for 5 min. Then the supernatant was carefully removed. Each pellet was resuspended in 600 µl of diluted serum (1:20) and incubated at 4°C on an overhead rotator for 2 h. The suspension was spun down (500 g, 3 min), the diluted serum supernatant removed and stored. Cells were washed two times with 1 ml 0.5% bovine serum albumin in PBS and two times with 1 ml physiological NaCl (Fresenius). After the last wash, cells were gently resuspended in 1 ml of elution buffer, placed on an overhead rotator for 3 min at RT and spun down at 300 g for 3 min. Immediately after, purified Ig containing eluates were neutralized with 1M Tris-HCl pH 9.5.

Isolation of specific SARS-CoV-2 N and canine distemper virus N antibodies (control) was performed according to manufacturers’ instructions. Briefly, 75 µl protein-coupled dynabeads (2 mg/ml) per antigen were added to the serum dilution and incubated for 60 min on a rotator at RT. After the incubation, diluted serum supernatants were removed and stored. Beads were washed three times with 1 ml 0.1% bovine serum albumin in PBS followed by one wash with physiological NaCl (Fresenius), each for 10 min on a rotatory shaker (RT). Elution of bound antibodies was performed with 300 µl of elution buffer (0.1M glycine in physiological NaCl, pH 2.5) for three minutes on a rotator, followed by 2 min on the magnet. Then, the supernatant containing purified Ig was immediately neutralized in 1M Tris pH 9.6.

In addition to the depletion methods mentioned above, quenching experiments were performed with one selected serum sample, using the following soluble recombinant SARS-CoV-2 proteins (all Genscript, Rijswijk, the Netherlands): S1 wildtype variant (Z03485-100), S1 delta variant B.1.617.2 (Z03612-100), S1 omicron variant B.1.1.529 (Z003729-100) and N omicron variant B.1.1.529 (Z03731-100) and in a separate experiment with soluble nucleocapsid proteins SARS-CoV2-N (wildtype, Invitrogen) and CoV-229E N (Sino Biologicals), respectively. Serum samples were diluted in 9.3% low IgG FCS in PBS and split into reaction tubes, each containing a different protein or PBS as untreated control resulting in a concentration of serum 1:100 in 10% FCS-PBS and 10µg protein/ml. Incubation was performed overnight at 4°C on a rotatory shaker. Samples were spun down (15 min, 20 000g) and quenched supernatants stored in aliquots at −20°C pending analysis by CBA and ELISA.

### Statistical analysis

For statistical analyses, the software packages GraphPad Prism (GraphPad Software, La Jolla, CA, USA; version 10) and IBM SPSS (IBM SPSS Statistics, Armonk, NY, USA; version 28) were used. The primary hypothesis of this study was that antibody responses to MOG and the occurrence of MOGAD are influenced (i) by SARS-CoV-2 infection and/or vaccination and (ii) by antibody responses against SARS-CoV-2 and other coronaviruses. This hypothesis was tested for antibody titres using between group comparisons of antibody levels by the Kruskal-Wallis test with Dunn’s multiple comparison test. Effect sizes of Kruskal-Wallis tests are shown as ε^2^ and *P*-values from Dunn’s multiple comparison test were corrected for multiple comparisons using a false discovery rate significance criterion of 1% based on the Benjamini, Krieger and Yekutieli correction. Principal component analysis was used to classify groups of log2-transformed antibody titres (CBA) and log10-transformed total antibody levels (ProcartaPlex assay). This unsupervised, unbiased multivariate analysis approach was used to explore the relation of antibody responses to MOG, SARS-CoV-2 and other coronaviruses. Loading plots were generated to visualize the combination antibody responses responsible for clustering. Correlations of antibody responses were evaluated by Spearman’s rank correlation tests. The predictive role of antibody response to SARS-CoV-2 and other coronaviruses, acute infections (SARS-CoV-2 or other infections), inflammatory demyelination, sex and age for MOG-IgG, MOG-IgA and MOG-IgM titres was analysed using ordinal regression analysis.

## Results

### Cross-reactivity of affinity-purified MOG antibodies to SARS-CoV-2 N and S proteins is a rare finding

**Figure 1:**
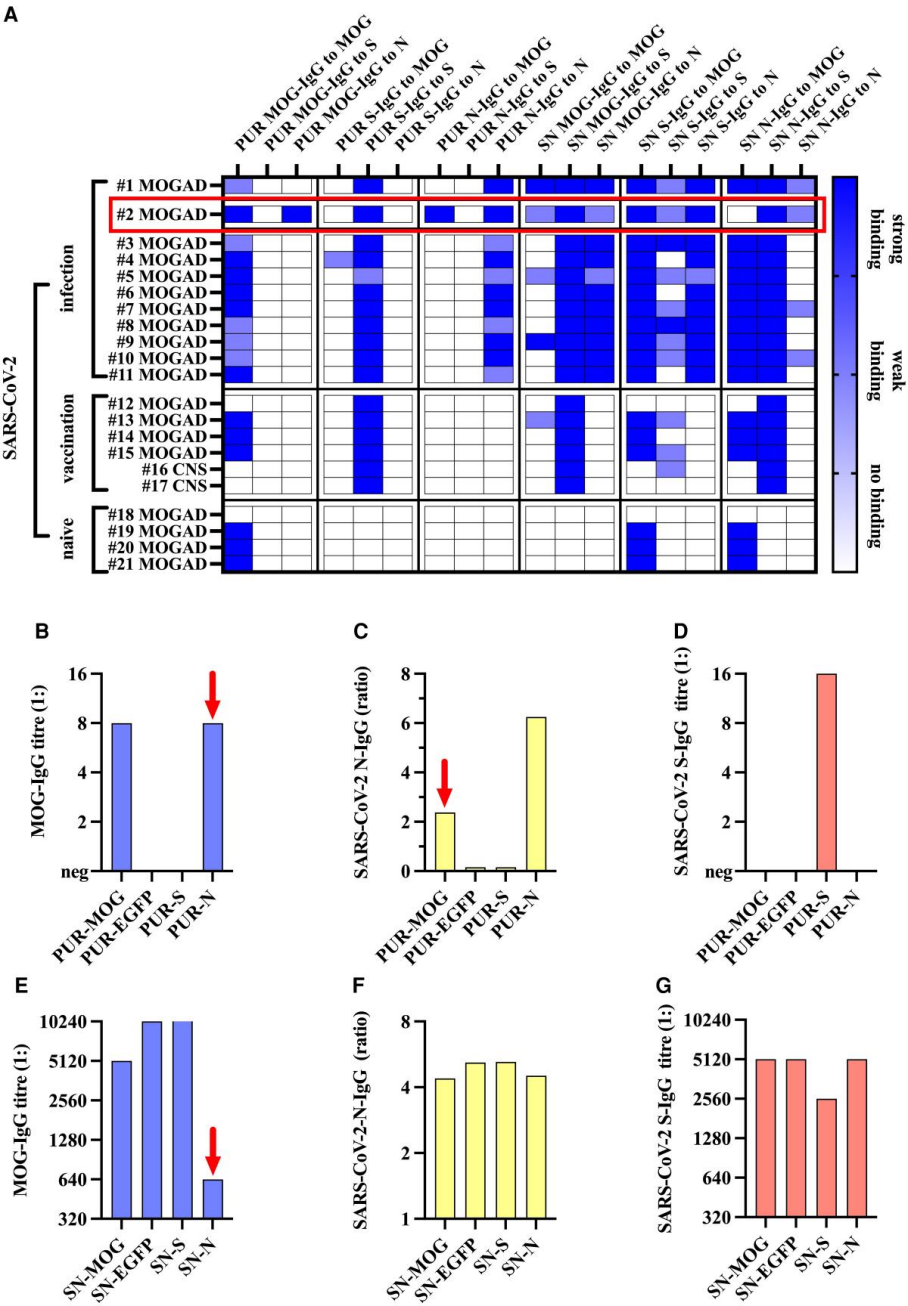
**Binding of affinity-purified antibodies to MOG and SARS-CoV-2 S and N proteins and antigen-specific depletion of antibodies**. (**A**) Heatmap showing the binding of affinity-purified antibodies to MOG and SARS-CoV-2 S and N proteins (PUR) or the unbound fraction after pre-incubation with MOG, S, N or EGFP, respectively (SN). Isolation of MOG-IgG was not successful in two MOGAD samples with low MOG-IgG titres (sample Nos 12 and 18). Two samples with cross-reactive purified antibodies were observed (sample Nos 2 and 4): sample No. 2 (MOGAD after COVID-19) showed a strong immunological cross-reactivity between MOG and SARS-CoV-2 N protein (highlighted by the red rectangle); sample No. 4 showed only very weak binding to MOG in CBA with purified S-IgG but no binding of purified MOG-IgG to SARS-CoV-2-S. Colour intensities indicate the degree of reactivity to the three antigens. Antibodies were affinity-purified using HEK293 cells expressing MOG, SARS-CoV-2 S or EGFP as control (data not shown), and SARS-CoV-2 N proteins coupled to magnetic beads. The purified antibody fractions and the respective supernatants after pre-incubation were then analysed for their binding to MOG in CBA or to SARS-CoV-2 S and N proteins in CBA and/or ELISA. Cross-reactive sample No. 2 (including EGFP as a control) is presented in more detail in (B-G), showing clear cross-reactive binding of purified N-IgG to MOG (**B**), cross-reactive binding of purified MOG-IgG to N (**C**) and no cross-reactivity of the two antibodies to SARS-CoV-2 S (**D**). Testing of SN showed strong depletion of MOG-IgG by SARS-CoV-2 N protein (**E**), strong depletion of N-IgG by MOG (at an equivalent degree to depletion by N protein) (**F**), and no depletion of SARS-CoV-2 S-IgG by N and MOG but only by S (**G**). Immunological cross-reactivity is indicated by red arrows. CNS, other demyelinating disease; EGFP, enhanced green fluorescent protein; IgG, immunoglobulin G; MOG, HEK293 cells expressing myelin oligodendrocyte glycoprotein (MOG); MOGAD, MOG antibody-associated demyelinating disease; N, recombinant SARS-CoV-2 nucleocapsid protein; PUR, affinity-purified antibodies; SARS-CoV-2, severe acute respiratory syndrome coronavirus 2; S, HEK293 cells expressing SARS-CoV-2 spike protein; SN, upernatant of affinity purification (not bound).

First, we affinity-purified MOG, SARS-CoV-2 S and SARS-CoV-2 N protein-specific antibodies from serum samples of patients with MOGAD after SARS-CoV-2 infection (n = 11) or vaccination (*n* = 4) and six controls (four MOGAD before the SARS-CoV-2 pandemics and two MOG-IgG-negative demyelination cases after SARS-CoV-2 vaccination (details are shown in Table 1 and Supplementary Table 1). As can be seen in Fig. 1A, successful purification of antigen-specific IgG was generally titre dependent, resulting in positive detection of purified MOG-IgG in most MOGAD patients (samples 1–11, 13–15, 19–21) except in two patients (samples 12 and 18), whereas all purified SARS-CoV-2 S-IgG (infected and vaccinated cases, samples 1–17) and SARS-CoV-2 *N*-IgG (infected cases, samples 1–11) from positive serum samples showed signals in both, CBA and ELISA. As expected, purification of specific antibodies was negative (as demonstrated by both CBA and ELISA) for patients without the respective antibodies present in serum. Analysis of the supernatants obtained after incubation with the transfected HEK293 cells or dynabeads revealed specific partial to complete depletion of the respective antigen-specific antibodies and confirmed our results, both in CBA and ELISA.

Cross-reactivity between purified SARS-CoV-2 S-IgG and MOG-IgG was generally not observed, except for one case (sample 4) with a very weak signal in MOG-IgG CBA at the starting dilution of 1:20. However, no reduction of MOG titres was observed in SARS-CoV-2 S-IgG-depleted supernatant of this sample. Furthermore, we observed no binding of any purified MOG-IgG fraction to SARS-CoV-2 S (CBA and ELISA), including sample 4.

**Figure 2:**
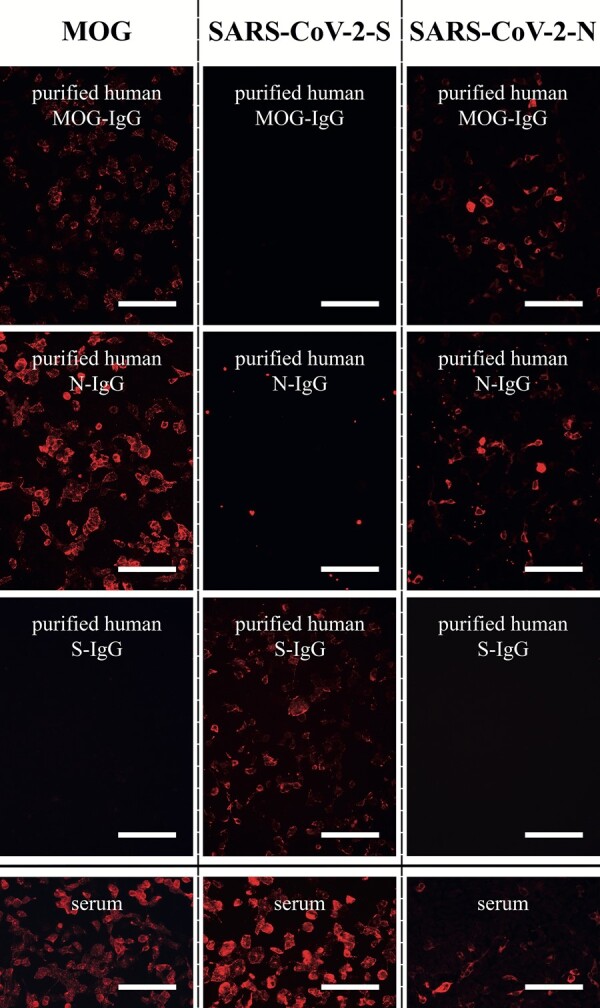
**Cell-based immunofluorescence assay demonstrating immunological cross-reactivity between MOG and SARS-CoV-2 N protein**. Purified human MOG-IgG (upper first panel), SARS-CoV-2 N-IgG (upper second panel), SARS-CoV-2 S-IgG (upper third panel) and serum samples from a patient (#2) with MOGAD post-COVID-19 were analysed for their reactivity to HEK293 cells transfected with human MOG, SARS-CoV-2 S and N. Bound human IgG antibodies were detected using an Alexa 594-fluorochrome labelled anti-human IgG (Fc-specific) secondary antibody. Please note unspecific signals of some transferred magnetic beads in panel ‘purified human N-IgG’ staining to SARS-CoV-2-S. Scale bar = 100 µm. IgG , immunoglobulin G; MOG, myelin oligodendrocyte glycoprotein; MOGAD, MOG antibody-associated demyelinating disease; N, SARS-CoV-2 nucleocapsid protein; SARS-CoV-2, severe acute respiratory syndrome coronavirus 2; S, SARS-CoV-2 spike protein.

Affinity-purified SARS-CoV-2 N-IgG showed no cross-reactivity to MOG and vice versa in samples 1 and 3–21. However, purified N-IgG of sample 2 showed a strong signal in MOG-IgG CBA (Fig. 1B, Fig. 2), and purified MOG-IgG showed strong binding in SARS-CoV-2 N-IgG ELISA (Fig. 1C) and CBA (Fig. 2). Cross-reactivity was confirmed by the reduction of MOG-IgG titres in SARS-CoV-2 N-IgG-depleted supernatant (Fig. 1E) and reduction in SARS-CoV-2 N-IgG ELISA ratios in MOG-IgG-depleted supernatant (Fig. 1F). Neither of the two antibody fractions (N-IgG, MOG-IgG) showed any signal against SARS-CoV-2 S in CBA (Fig. 1D, Fig. 2) or ELISA (data not shown), and, conversely, SARS-CoV-2 S-IgG-depleted supernatants did not show decrease of MOG-IgG titres (Fig. 1E) or SARS-CoV-2 N-IgG ELISA ratios (Fig. 1F).

To exclude unspecific binding of serum antibodies to either transfected HEK293 cells or magnetic beads during the antibody purification process, all samples were also affinity purified with enhanced green fluorescent protein-transfected HEK293 cells and canine distemper virus N protein bound to magnetic beads. All control affinity-purified fractions were negative in all CBA and ELISA used for analysis (Fig. 1B–G for sample 2, other data not shown) and supernatants showed no loss or depletion of the antibodies analysed and hence served as control reference (Fig. 1E–G for sample 2, other data not shown).

Antibody binding to SARS-CoV-2 S protein in ELISA was reduced after pre-absorption with S proteins in all SARS-CoV-2 infected or vaccinated samples. Antibody binding to SARS-CoV-2 N in ELISA was also reduced after pre-absorption with N protein. Pre-absorption with S proteins did not affect antibody binding to N protein and vice versa.

### Characterisation of the immunological cross-reactivity between MOG and SARS-CoV-2 n protein

Sample No. 2 which showed antibody cross-reactivity between MOG and SARS-CoV-2 N protein was from a 20-year-old unvaccinated female patient who presented with encephalomyelitis after SARS-CoV-2 infection (2021, PCR confirmed infection). She had a history of fever and upper respiratory COVID-19 symptoms responsive to symptomatic treatment (with good recovery) 2 weeks before the onset of neurological symptoms and no evidence of other infections or neurological/systemic disorders. The patient was treated with intravenous corticosteroids and had a monophasic disease course so far (follow-up 10 months), with good recovery. MOG-IgG titre at baseline was high (1:20,480) and declined to 1:80 at follow-up (seronegative) after 1 year. Interestingly, this patient was also seropositive for total Ig antibodies against CoV-229E (13,285 U/ml), CoV-HKU1 (17 951 U/ml), CoV-OC43 (13 888 U/ml) and CoV-NL63 (11 388 U/ml). Moreover, this sample showed binding of IgG to SARS-CoV-2 N and CoV-229E N proteins in ELISA (Supplementary Fig. 3). We have therefore analysed whether purified MOG antibodies and SARS-CoV-2 N antibodies of this patient were also cross-reactive with CoV-229E N protein. Additionally, depletion/quenching experiments with MOG-expressing cells and soluble COV-229E N and SARS-CoV-2 N protein were performed. We could not find any reduction of MOG-IgG titres after pre-incubation with CoV-229E N protein or binding of purified MOG-IgG to CoV-229E (Supplementary Fig. 3), whereas a reduction of MOG-IgG titres after pre-incubation with SARS-CoV-2 N was observed as expected. However, purified SARS-CoV-2 N-IgG showed weak cross-reactivity to CoV-229E N, which conversely could be confirmed by a reduction of antibody binding to CoV-229E N in SARS-CoV-2 N-IgG-depleted supernatants (Supplementary Fig. 3). Finally, we excluded a cross-reactivity between MOG and variant-specific S proteins by pre-incubation with SARS-CoV-2 S proteins from delta and omicron variants (Supplementary Fig. 4).

A homology search with BLAST showed no significant sequence homologies between MOG and SARS-CoV-2 N or CoV-229E N protein. Moreover, ELISA analysis of the cross-reactive case by using a library of overlapping SARS-CoV-2 N peptides could not determine significant binding to any linear epitope (Supplementary Fig. 5).

### Association of antibodies against MOG, SARS-CoV-2 and other coronaviruses

**Figure 3:**
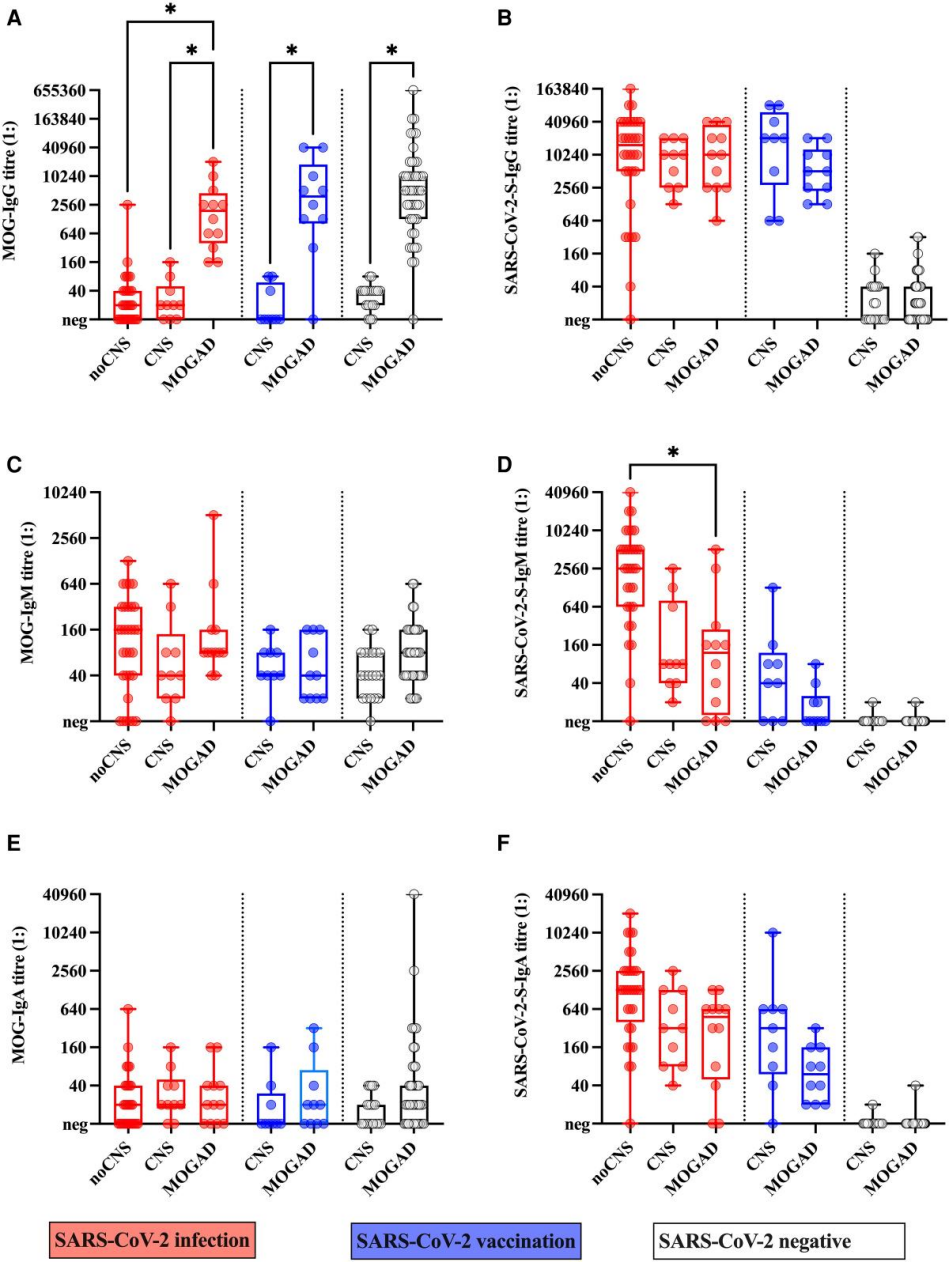
**Antibody reactivity against MOG and SARS-CoV-2 S in samples from individuals with SARS-CoV-2 infection (COVID-19), SARS-CoV-2 vaccination or negative/naïve for SARS-CoV-2, as measured by CBA**. (**A**) IgG (*P* < 0.001, ε^2^ = 0.685), (**C**) IgM (*P* = 0.080, ε^2^ = 0.082) and (**E**) IgA (*P* = 0.220, ε^2^ = 0.059) antibodies against MOG were measured using a live CBA with MOGα1-transfected HEK293 cells. (**B**) IgG (*P* < 0.001, ε^2^ = 0.729), (**D**) IgM (*P* = 0.001, ε^2^ = 0.788) and (**F**) IgA (*P* < 0.001, ε^2^ = 0.811) antibodies against SARS-CoV-2 S protein were measured using a live CBA with SARS-CoV-2 spike protein (S)-tr**a**nsfected HEK293 cells. Significance of group differences was tested using the Kruskal-Wallis test and effect sizes ε^2^ and *P*-values are indicated above. Only group differences (analysed Dunn’s multiple comparison test) within each subgroup (SARS-CoV-2 infection, SARS-CoV-2 vaccination or negative/naïve) are shown, asterisks indicate a significant difference between groups after correction for multiple comparisons (21) using the two-stage linear step-up procedure of Benjamini, Krieger and Yekutieli. Data are shown as boxplots (boxes represent 25th to 75th percentiles with medians indicated by the horizontal lines, whiskers represent minimum to maximum) with individual data points (circles). CNS, other demyelinating disease; IgA, immunoglobulin A; IgG, immunoglobulin G; IgM, immunoglobulin M; MOG, myelin oligodendrocyte glycoprotein; MOGAD, MOG antibody-associated demyelinating disease; noCNS, no neurological symptoms; SARS-CoV-2, severe acute respiratory syndrome coronavirus 2; S, SARS-CoV-2 spike protein.

**Figure 4:**
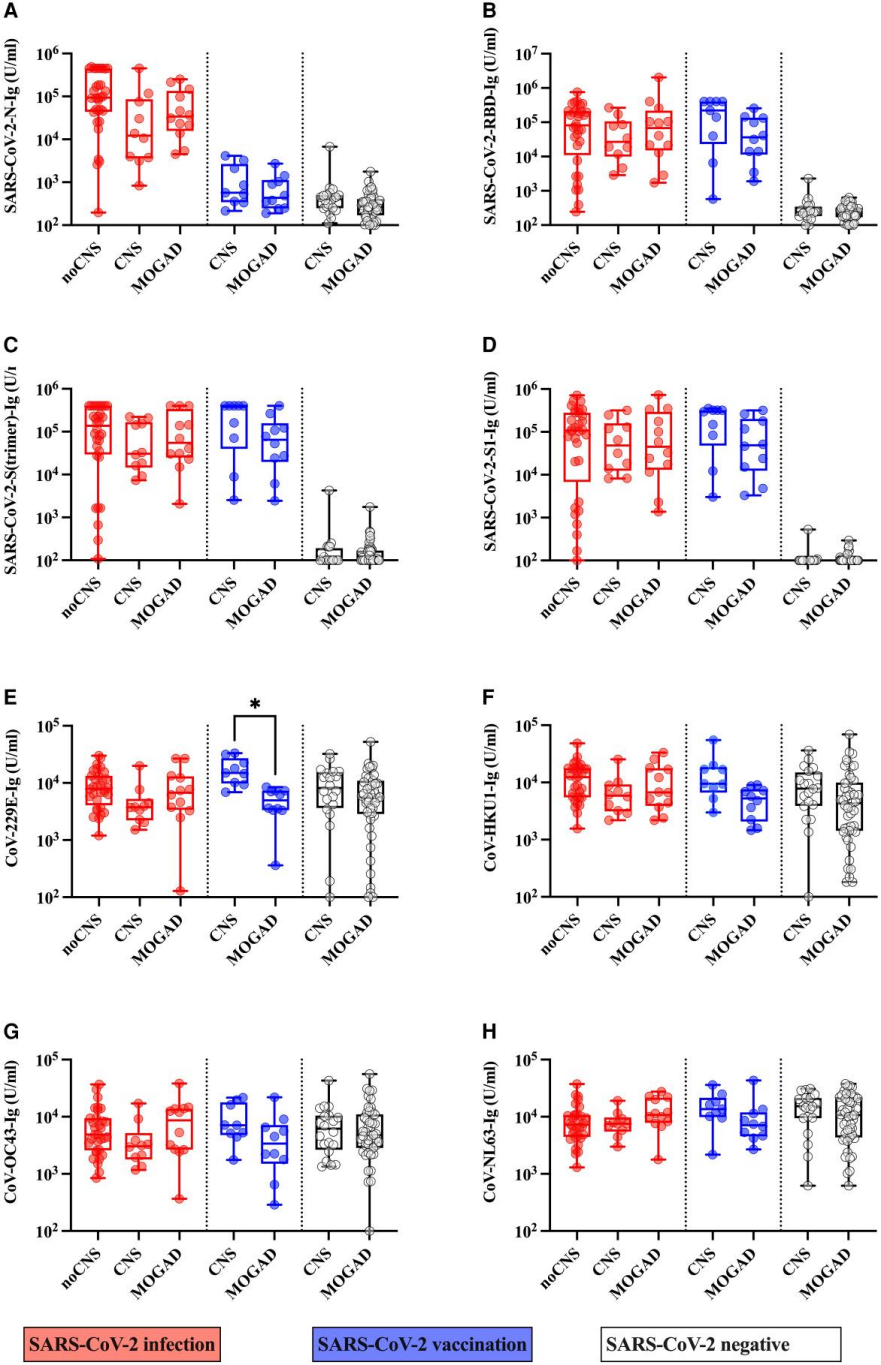
**Antibody reactivity against SARS-CoV-2 and common coronaviruses in samples from people with SARS-CoV-2 infection (COVID-19), SARS-CoV-2 vaccination or negative/naïve for SARS-CoV-2, as measured by ProcartaPlex assay**. Total immunoglobulin (Ig) antibodies against (**A**) SARS-CoV-2 N (*P* < 0.001, ε^2^ = 0.697), (**B**) RBD (*P* < 0.001, ε^2^ = 0.725), (**C**) S(trimer) (*P* < 0.001, ε^2^ = 0.732) and (**D**) S1 proteins (*P* < 0.001 ε^2^ = 0.737); and (**E**) CoV-229E (*P* = 0.007 ε^2^ = 0.128), (**F**) CoV-HKU1 (*P* = 0.008, ε^2^ = 0.126), (**G**) CoV-OC43 (*P* = 0.330 ε^2^ = 0.049) and (**H**) CoV-NL63 (*P* = 0.090, ε^2^ = 0.079) were measured using the Coronavirus Ig Total Human 11-Plex ProcartaPlex™ Panel (Invitrogen, EPX110-16000-901). Significance of group differences was tested using the Kruskal-Wallis test and effect sizes ε^2^ and *P*-values are indicated above. Only group differences (analysed Dunn’s multiple comparison test) within each subgroup (SARS-CoV-2 infection, SARS-CoV-2 vaccination or negative/naïve) are shown, asterisks indicate a significant difference between groups after correction for multiple comparisons (21) using the two-stage linear step-up procedure of Benjamini, Krieger and Yekutieli. Data are shown as boxplots (boxes represent 25th to 75th percentiles with medians indicated by the horizontal lines, whiskers represent minimum to maximum) with individual data points (circles). CNS, other demyelinating disease; CoV, coronavirus; Ig, total immunoglobulin; IgA, immunoglobulin A; IgG, immunoglobulin G; IgM, immunoglobulin M; MOG, myelin oligodendrocyte glycoprotein; MOGAD, MOG antibody-associated demyelinating disease; N, SARS-CoV-2 nucleocapsid protein; noCNS, no neurological symptoms; RBD, receptor binding domain; SARS-CoV-2, severe acute respiratory syndrome coronavirus 2; S, SARS-CoV-2 spike protein; S1, S1 subunit SARS-CoV-2 spike protein; S(trimer), trimer of SARS-CoV-2 spike protein; U/ml, U/ml.

Based on our observations, we next analysed the relation of antibody responses against MOG (IgG, IgM, IgA), SARS-CoV-2 (S, S1, S(trimer), RBD and N; IgG, IgM, IgA or total Ig) and other coronaviruses (CoV-229E, CoV-HKU-1, CoV-OC43 and CoV-NL63; total Ig) in serum samples of patients with SARS-CoV-2 infection and neurological symptoms with (n = 12) or without MOG-IgG (*n* = 10); SARS-CoV-2 infection without neurological symptoms (*n* = 32); SARS-CoV-2 vaccination and neurological symptoms with (*n* = 10) or without MOG-IgG (*n* = 9); and SARS-CoV-2 negative/naïve patients with neurological symptoms with (*n* = 47) or without MOG-IgG (*n* = 20) using an antibody profiling approach. As expected, MOG-IgG (Fig. 3A) antibodies were highest in MOGAD patients, whereas MOG-IgM (Fig. 3C) and MOG-IgA (Fig. 3E) titres did not differ between groups. No differences between groups with a history of SARS-CoV-2 infection or vaccination were observed in the distribution of IgG or IgA antibodies against SARS-CoV-2 S (Fig. 3B and F). However, among patients with a history of SARS-CoV-2 infection, SARS-CoV-2 S IgM titre levels were significantly lower in MOGAD cases than in patients without neurological symptoms (Fig. 3D). Overall, our results indicate no significant differences between individuals with or without MOG-IgG for antibody responses against SARS-CoV-2 and other coronaviruses (Fig. 4) with the exception of significantly higher CoV-229E antibody levels in neurological cases without MOG-IgG compared to MOG-IgG positive cases in the subgroup of patients vaccinated against SARS-CoV-2 (Fig. 4E).

**Figure 5:**
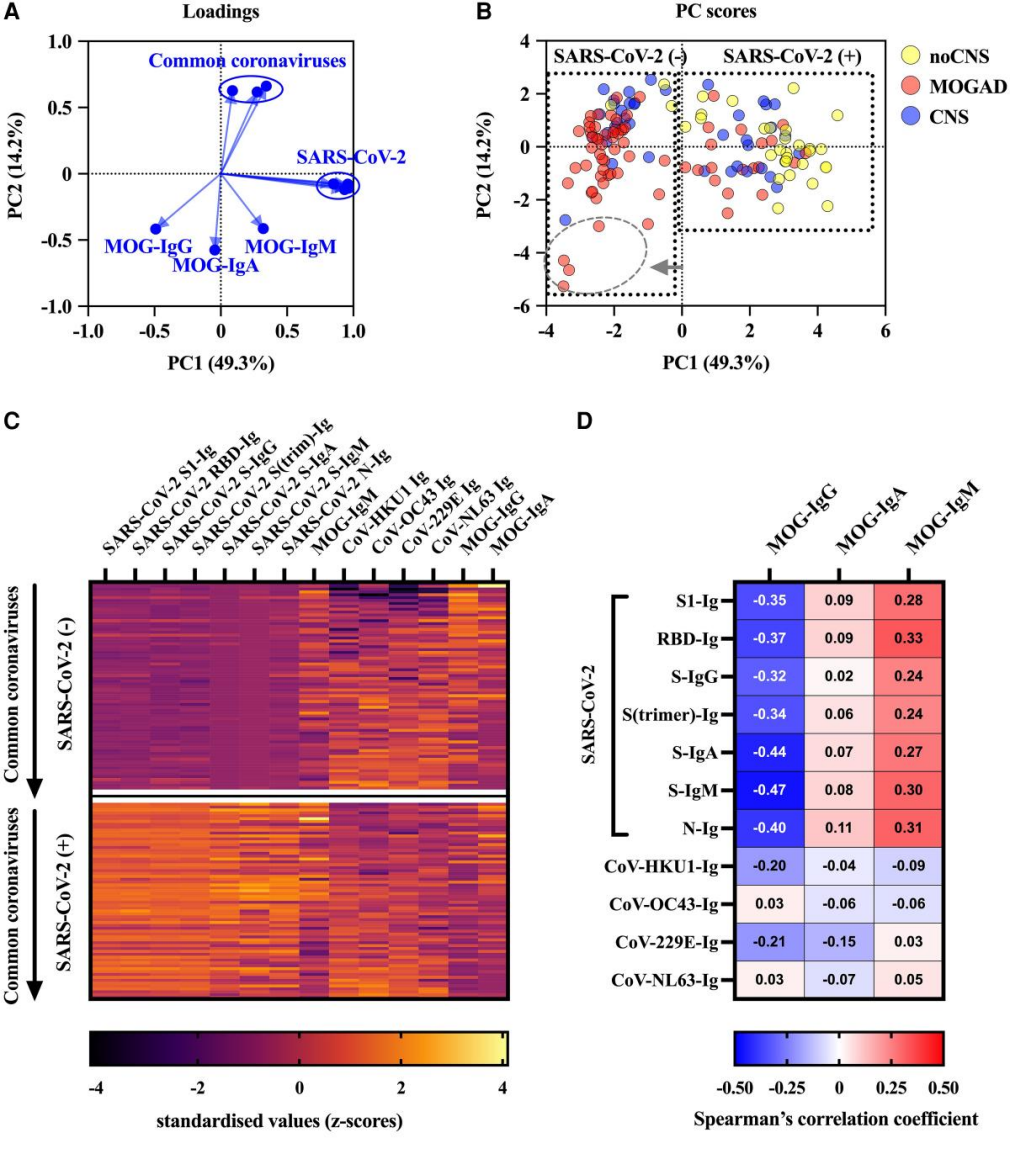
**Relation of antibody reactivities against MOG, SARS-CoV-2 S and N proteins and common coronaviruses (CoV-229E, CoV-HKU1, CoV-OC43 and CoV-NL63) analysed by principal component analysis**. (**A**) Scatter dot plot showing the loading scores of all antibodies analysed to visualize parameters responsible for clustering. (**B**) Principal component (PC) scores of all samples. A subgroup of samples with high MOG-IgG titres but low/negative antibody levels against SARS-CoV-2 and common coronaviruses is indicated by a circle and a grey arrow. (**C**) Heatmap of the quantitative results (standardized values of log-transformed antibody levels) according to their PC1 and PC2 scores. (**D**) Spearman correlation coefficients of MOG antibodies (IgG, IgA and IgM) with antibodies against SARS-CoV-2 and common coronaviruses. CNS, other demyelinating disease; CoV, coronavirus; IgA, immunoglobulin A; Ig, total immunoglobulin; IgG, immunoglobulin G; IgM, immunoglobulin M; MOG, myelin oligodendrocyte glycoprotein; MOGAD, MOG antibody-associated demyelinating disease; N, SARS-CoV-2 nucleocapsid protein; noCNS, no neurological symptoms; PC, principal component; RBD, receptor binding domain; SARS-CoV-2, severe acute respiratory syndrome coronavirus 2; SARS-CoV-2 (−), SARS-CoV-2 antibody negative; SARS-CoV-2 (+), SARS-CoV-2 antibody positive; S, SARS-CoV-2 spike protein; S1, S1 subunit SARS-CoV-2 spike protein; S(trimer), trimer of SARS-CoV-2 spike protein.

We have then used principal component analysis to explore the relationship of the different antibodies to MOG, SARS-CoV-2 and other coronaviruses and to classify groups in an unbiased approach. As can be seen in Fig. 5, antibody responses against SARS-CoV-2 (PC1) and other coronaviruses (PC2) were clustered and associated, but distinct from antibody responses against MOG, with the exception of MOG-IgM being associated with SARS-CoV-2 antibodies (Fig. 5A). The PC plot and the heatmap of this reduction to two dimensions (PC1 and PC2) show the presence of antibody responses against SARS-CoV-2 (PC1) to be the main difference, whereas antibody responses against other coronavirus and MOG were similar in both groups (Fig. 5B, Fig. 5C). Importantly, there was a subgroup of (very young) patients with high MOG-IgG titres but absent antibodies to any coronavirus. Overall, MOG-IgG antibodies were negatively correlated with antibodies against SARS-CoV-2 or other coronavirus, whereas MOG-IgM titres were positively correlated with antibodies against SARS-CoV-2 (Fig. 5D).

Next, we analysed the predictive role of antibody response to SARS-CoV-2 and other coronaviruses, acute infections (SARS-CoV-2 or other infections), inflammatory demyelination, sex and age on MOG antibody classes using ordinal regression analysis (Table 2). MOG-IgG antibody titres were significantly lower in females than in males and significantly higher in individuals with inflammatory demyelinating syndromes than in those without. Interestingly, MOG-IgG levels were significantly higher after non-COVID infections than after COVID-19. Furthermore, both MOG-IgG and MOG-IgA titres had a negative moderate correlation with CoV-229E antibodies. MOG-IgM antibody titres were significantly higher in females than in males and showed a moderate positive correlation with SARS-CoV-2 *N*-Ig antibody levels, but not with the antibody levels to the other coronaviruses. Importantly, neither MOG-IgM nor MOG-IgA antibody titres were specifically associated with inflammatory demyelinating syndromes but were also found in patients without neurological symptoms.

**Table 2:** Ordinal regression analysis for predictors for MOG antibody responses

	MOG-IgG titre (1:)^a^	MOG-IgM titre (1:)^b^	MOG-IgA titre (1:)^c^
Females	−0.78 (−1.41 to −0.15)*	0.67 (0.04 to 1.31)*	−0.29 (−0.94 to −0.36)
Children (age < 18 years)	−0.07 (−0.85 to 0.71)	−0.07 (−0.86 to 0.73)	−0.74 (−1.58 to 0.09)
Inflammatory demyelination	2.05 (1.00 to 3.12)***	0.44 (−0.57 to 1.44)	0.99 (−0.06 to 2.05)
Acute infection			
Other infection	1.60 (0.63 to 2.56)**	0.75 (−0.20 to 1.70)	0.89 (−0.07 to 1.86)
SARS-CoV-2 (COVID-19)	−0.22 (−1.58 to 1.15)	−1.20 (−2.58 to 0.19)	−0.66 (−2.11 to 0.79)
SARS-CoV-2-N-Ig	0.75 (−0.20 to 1.70)	1.17 (0.53 to 1.81)***	0.68 (0.02 to 1.34)*
SARS-CoV-2-S1-Ig	−1.20 (−2.58 to 0.19)	0.51 (−0.19 to 0.39)	−0.01 (−0.312 to 0.29)
CoV-229E-Ig	0.89 (−0.07 to 1.86)	0.10 (−0.58 to 0.77)	−0.88 (−1.58 to −0.18)*
CoV-HKU-Ig	−0.66 (−2.11 to 0.79)	−0.47 (−1.19 to 0.25)	−0.56 (−1.30 to 0.18)
CoV-OC43-Ig	0.39 (−0.23 to 1.01)	1.17 (0.53 to 1.81)***	0.68 (0.02 to 1.34)*
CoV-NL63-Ig	−0.21 (−0.50 to 0.07)	0.51 (−0.19 to 0.39)	−0.01 (−0.312 to 0.29)

Data are shown as regression coefficients b with 95% CIs.

Model fit:^a^MOG-IgG: Cox&Snell *R*^2^ = 0.401, Nagelkerke *R*^2^ = 0.403, McFadden *R*^2^ = 0.102, chi-square = 71.640, *P* < 0.001.

^b^MOG-IgM: Cox&Snell *R*^2^ = 0.217, Nagelkerke *R*^2^ = 0.222, McFadden *R*^2^ = 0.066, chi-square = 34.209, *P* < 0.001.

^c^MOG-IgA: Cox&Snell *R*^2^ = 0.127, Nagelkerke *R*^2^ = 0.133, McFadden *R*^2^ = 0.044, chi-square = 19.039, *P* = 0.060.

*P < 0.05, **P < 0.01, ***P < 0.001.

CoV, coronavirus; COVID-19, coronavirus disease 2019; Ig, immunoglobulin; IgG, immunoglobulin G; MOG, myelin oligodendrocyte glycoprotein; N, SARS-CoV-2 nucleocapsid protein; SARS-CoV-2, severe acute respiratory syndrome coronavirus; S, SARS-CoV-2 spike protein.

### Different temporal dynamics of antibodies against MOG, SARS-CoV-2 and other coronaviruses

**Figure 6:**
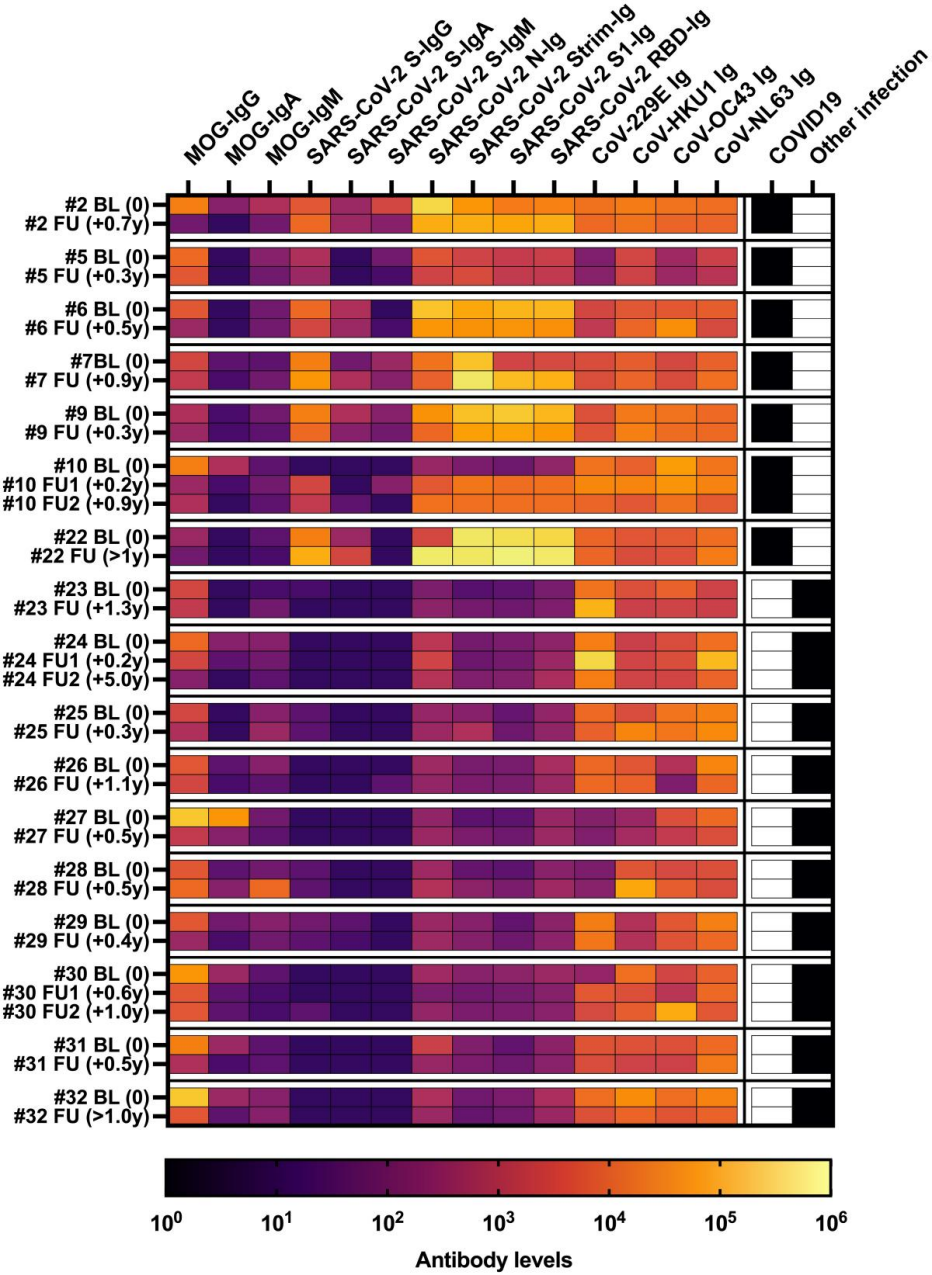
**Different temporal dynamics of antibodies against MOG, SARS-CoV-2 and common coronaviruses**. Heatmap of follow-up samples of 17 MOGAD patients (7 with COVID-19 and 10 with other infections). Colour intensity indicates the levels (titres or U/ml) of the measured antibodies. Most samples showed a differential change (decrease versus increase and vice versa) of antibodies against MOG, SARS-CoV-2 and other coronaviruses. Sample No. 2 is from the patients with cross-reactive antibodies. BL, baseline; CoV, coronavirus; COVID-19, coronavirus disease 2019; FU, follow-up with follow-up time; Ig, total immunoglobulin; IgA, immunoglobulin A; IgG, immunoglobulin G; IgM, immunoglobulin M; MOG, myelin oligodendrocyte glycoprotein; N, SARS-CoV-2 nucleocapsid protein; RBD, receptor binding domain; SARS-CoV-2, severe acute respiratory syndrome coronavirus; S, SARS-CoV-2 spike protein; S1, S1 subunit SARS-CoV-2 spike protein; S(trimer), trimer of SARS-CoV-2 spike protein; y, years.

Finally, we analysed follow-up samples of 17 MOGAD patients [7 with COVID-19 and 10 pre-pandemic cases with other (unknown) infections]. All baseline samples were obtained at the time of neurological onset to compare the temporal patterns of antibody responses against MOG, SARS-CoV-2 and other coronaviruses (Fig. 6). Most patients showed a decrease of MOG-IgG and MOG-IgA titres in follow-up samples. In contrast, antibody levels against SARS-CoV-2 and other coronaviruses remained relatively stable in most patients, and patient no. 10 showed an increase of SARS-CoV-2 antibodies indicative of a confirmed infection with SARS-CoV-2 after initial presentation with MOGAD. A follow-up sample (8 months) of the MOGAD patient with antibody cross-reactivity (no. 2) showed a decrease in MOG-IgG antibody levels compared to the first event, whereas antibody levels against SARS-CoV-2 and other coronaviruses remained relatively stable.

## Discussion

Many studies indicate that the onset of MOGAD is associated with a broad range of infections and vaccinations.^23,24^ Furthermore, recent studies suggested MOGAD to be a rare neurological post-acute sequelae after SARS-CoV-2 infection or vaccination, pointing to a possible role of both as a potential trigger of MOGAD.^7-22^ In this study, we therefore aimed to elucidate whether there is an immunological cross-reactivity between SARS-CoV-2 S or N proteins and MOG and to explore the relation of antibody responses against MOG and SARS-CoV-2 and other coronaviruses. To the best of our knowledge, this is the first report demonstrating an immunological cross-reactivity of human autoantibodies directed against MOG and a viral nucleocapsid protein in a patient with MOGAD occurring after COVID-19. This cross-reactivity was shown by (i) the binding of affinity-purified human MOG antibodies to SARS-CoV-2 N protein in CBA and ELISA, (ii) the binding of affinity-purified human SARS-CoV-2 N antibodies to MOG in CBA and (iii) pre-adsorption of human serum with soluble SARS-CoV-2 N protein reducing binding to MOG and, vice versa, pre-adsorption with MOG reducing binding to SARS-CoV-2 N. Since we were not able to identify a linear SARS-CoV-2 N peptide recognized by the purified MOG antibody fraction or serum, we assume that a conformational epitope on both MOG and SARS-CoV-2 N is responsible for this cross-reactivity. Interestingly, this patient was also seropositive for other coronaviruses (CoV-229E, CoV-HKU1, CoV-OC43 and CoV-NL63), and purified antibodies against SARS-CoV-2 N also recognized CoV-229E N protein. Although we were not able to detect clear cross-reactivity of purified MOG antibodies and CoV-229E N protein, it is tempting to speculate that a previous infection with CoV-229E may have generated potentially cross-reactive antibodies interacting weakly with MOG, which then were reactivated after infection with SARS-CoV-2. This mechanism is also called ‘antigenic sin’, in which previous immune responses to viruses might shape subsequent responses to other related viruses and has been observed after infection with influenza virus,^34^ human immunodeficiency virus^35^ and most recently also SARS-CoV-2.^22,36^ Specifically, Spatola et al.^22^ could demonstrate an expanded immune response against common coronaviruses (CoV-229E, CoV-NL63 and CoV-OC43) in patients with neuroPASC after SARS-CoV-2 infection. These findings indicate that, after encountering a pathogen for the second time, the initial immune response influences the secondary response and may thereby trigger autoimmunity to MOG and other CNS proteins. However, since no sample from before the onset of COVID-19/MOGAD was available in the patient with cross-reactive antibodies, we were not able to confirm the presence of CoV-229E antibodies from before infection with SARS-CoV-2 to support our hypothesis.

We could only investigate a small number of MOGAD cases with SARS-CoV-2 infection due to the rarity of this condition, and only one patient showed IgG cross-reactivity between MOG and SARS-CoV-2 N protein. Hence, it is unlikely that this immunological cross-reactivity fully explains the occurrence of MOGAD after COVID-19. Furthermore, recent findings do not indicate a significant increase in the incidence of MOGAD during the pandemic and MOGAD without COVID-19 occurs at the same frequency.^7,10,12,14,16,17,20^ Moreover, MOGAD also occurs after vaccination with SARS-CoV-2 S mRNA or, in particular, vector vaccines.^8,9,11,13,15,18,19,21^ All these reports are supported by our results in serological profiling of pre-pandemic and pandemic samples showing no or even negative correlation between the presence of MOG-IgG and antibodies to not only SARS-CoV-2 but also to CoV-229E, CoV-HKU1, CoV-OC43 and CoV-NL63. Moreover, follow-up samples also show no clear association of antibody levels to different coronaviruses and MOG-IgG titres. Like in other common infections, the frequency of coronavirus antibody detection rises with age. In this context, it is important to mention that we found a few very young MOGAD cases in our study who were seronegative for the coronaviruses analysed in our study. To conclude, although infection with coronaviruses may in rare cases trigger MOGAD, our data do not support a strong association of coronavirus infections and MOGAD.

In contrast to the lack of a positive correlation of MOG-IgG and coronavirus antibodies, we observed a non-significantly elevated level of MOG-IgA and a significant increase of MOG-IgM in SARS-CoV-2-infected individuals, whereas there were no associations with any other common coronavirus antibodies. Interestingly, this observation was present in COVID-19 patients of all investigated groups and hence independent of the presence of neurological symptoms (neuroPASC and MOGAD). This rather unspecific association of MOG-IgA and MOGAD is consistent with an older study by Pedreno *et al.*,^37^ whereas a recent study by Ayroza Galvão Ribeiro Gomes suggested that MOG-IgA may be a novel diagnostic biomarker for patients with CNS demyelination.^38^ The increased levels of MOG-IgM results are in line with reports on unspecific natural IgM autoantibody responses commonly observed after infection with SARS-CoV-2 and other viruses such as influenza.^39,40^ Finally, our observation might offer an explanation for the frequent but unspecific occurrence of MOG-IgM in neurological diseases and healthy individuals, owing to a preceding serious infection.^28,41,42^

Our study has a couple of limitations, the major limitation being the relatively low number of included patients which is due to the rarity of MOGAD after SARS-CoV-2 infections or vaccination. However, if the signal of a connection between SARS-CoV-2 infection and presence of MOG antibodies would have been overwhelming (>50%), the sample size included would already have exposed it at 5% alpha and 80% beta. Another limitation is age differences between groups, with COVID-19 patients being older than many controls. In contrast to COVID-19, we had no information on the cause of other infections and most importantly we could not confirm or exclude infections with other coronaviruses as the trigger of MOGAD. It should also be mentioned that the measurement of antibody levels of coronaviruses only indicates seroconversion and provides no or little information on the timepoint of infection. Moreover, four of the samples initially diagnosed with MOGAD after SARS-CoV-2 infection or vaccination at the initially testing centres were negative for MOG-IgG with our assay. This finding might be caused either by increased MOG-IgM having initially resulted in falsely positive MOG-IgG titres in assays using H + L-specific rather than Fc(gamma)-specific detection antibodies,^28,41^ by isolated CSF MOG-IgG but negative serum MOG-IgG positivity,^43,44^ by immunosuppressive treatments,^6,23^ the timing of sample collection in regard to the neurological presentation^45,46^ or by the degradation of antibodies due to repeated freeze-thaw cycles.^47,48^ This is important because the purification of antigen-specific antibodies is dependent on antibody titre and consequently was less efficient in samples with lower antibody levels to MOG or SARS-CoV-2 S and N proteins. However, only few (15%) of the included patients were on suppressive treatment at sampling with comparable frequencies in all groups (Table 1) and most samples (71%) were obtained within 3 months after the neurological event. Moreover, as can be seen from Supplementary Fig. 6, treatment or time from neurological event to sampling had no clear effect on antibody titres. The median number of freeze-thaw cycles was 2 (range 1–5) and therefore was in a range previously reported not affecting the stability of different autoantibodies or antibodies against SARS-CoV-2.^47,48^

Finally, the emergence of different SARS-CoV-2 variants, particularly the omicron variant, might have influence our results on the cross-reactivity of MOG and SARS-CoV-2 S antibodies. However, our findings indicate that this is not a problem for SARS-CoV-2 N protein which is much better conserved.

To conclude, although infection with coronaviruses may in rare cases trigger MOGAD and we have for the first time identified an immunological (i.e. IgG) cross-reactivity between MOG and a viral nucleocapsid protein), our data do not support a strong association of coronavirus infections and MOGAD. Furthermore, we provide first evidence how a viral infection could lead to the occurrence of MOG-IgG antibodies and hope that our study could provide important information for future studies on the role of infections in autoimmune responses to MOG.

## Supplementary Material

fcae106_Supplementary_Data

## Data Availability

The data supporting the findings of this study are available on request from the corresponding author (M.R.). The data are not publicly available due to ethical restrictions (data protection).
