# Supplementary material for: Is there an immunological cross-reactivity of antibodies to the myelin oligodendrocyte glycoprotein and coronaviruses?
Source: Brain Commun. 2024 Mar 25;6(2):fcae106. doi: 10.1093/braincomms/fcae106 (PMC10994262; doi:10.1093/braincomms/fcae106)
Supplement: fcae106_Supplementary_Data [file fcae106_supplementary_data.pdf]

SUPPLEMENTARY INFORMATION

Supplementary Table 1 Clinical and serological data of patient samples analysed for antibody cross-reactivity between MOG and SARS-CoV-2 nucleocapsid and spike proteins

Sample Nr. (year)	Age (y)/ Sex	Clinical presentation	MOG-antibodies (1:)			SARS-CoV-2 spike-antibodies (1:)			SARS-CoV-2 S1[a] IgG	SARS-CoV-2 N[b] IgG
			IgG	IgA	IgM	IgG	IgA	IgM		
**MOGAD after SARS-CoV-2 infection (COVID-19)**										
#1 (2021)	85/F	Encephalitis	160	40	5120	2560	640	5120	612.90	6.86
#2 (2021)	20/F	ADEM	20480	160	640	5120	640	2560	239.62	6.28
#3 (2021)	36/F	Myelitis	640	40	160	40960	1280	40	5307.11	4.80
#4 (2022)[c]	29/F	ON bilateral	2560	20	80	10240	40	20	596.35	3.20
#5 (2022)	7/M	ADEM	10240	<20	160	640	<20	80	48.28	0.60
#6 (2022)	13/F	NMOSD	5120	<20	80	10240	640	<20	1147.26	5.70
#7 (2021)[c]	28/M	ON bilateral	2560	40	40	20480	80	320	54.77	1.60
#8 (2023)	15/M	Cerebral	1280	<20	80	40960	320	<20	2559.56	3.30
#9 (2021)[c]	37/M	ON bilateral	640	20	80	20480	640	160	1344.19	3.50
#10 (2021)[c]	13/F	ADEM-ON	320	20	80	2560	<20	160	199.93	1.20
#11 (2021)	54/F	Brainstem	2560	160	80	2560	640	160	374.72	2.60
**MOGAD after SARS-CoV-2 vaccination**										
#12 (2021)	18/F	ON	<20	<20	160	5120	160	40	1190.75	0.14
#13 (2021)	17/M	ADEM	40960	160	80	2560	160	20	479.07	0.14
#14 (2021)	67/M	ON	1280	20	20	10240	20	<20	562.70	0.06
#15 (2921)	33/M	ON	5120	80	40	40960	1280	40	2531.10	0.20
**MOG-IgG-negative demyelination after SARS-CoV-2 vaccination**										
#16 (2021)	25/M	ADEM	<20	<20	80	5120	160	40	669.60	0.31
#17 (2021)	26/F	ON	40	<20	40	20480	640	<20	2980.99	0.23
**MOGAD before the COVID-19 pandemics**										
#18 (2018)	40/F	Myelitis	<20	<20	640	<20	<20	<20	6.73	0.39
#19 (2015)[c]	7/F	Myelitis	2560	40	80	<20	<20	40	7.50	0.20
#20 (2019)	54/F	NMOSD	10240	40	320	320	<20	<20	7.83	0.27
#21 (2018)	62/M	Encephalitis	5120	40	160	<20	<20	<20	8.00	0.15

[a]Antibody levels to SARS-CoV-2 spike (S1) protein in binding antibody units per millilitre (BAU/ml), [b]Antibody levels to SARS-CoV-2 nucleocapsid (N) protein in antibody indices, [c] Immunosuppressive treatment at sampling.

ADEM = acute disseminated encephalomyelitis, F = female, IgA = immunoglobulin A, IgG = immunoglobulin G, IgM = immunoglobulin M, M= male, MOG = myelin oligodendrocyte glycoprotein, MOGAD = MOG antibody associated disease, N = SARS-CoV-2 nucleocapsid protein, NMOSD = neuromyelitis optica spectrum disorder, ON = optic neuritis, SARS-CoV-2 = severe acute respiratory syndrome coronavirus, S1 = subunit 1SARS-CoV-2 spike protein.

Supplementary Figure 1

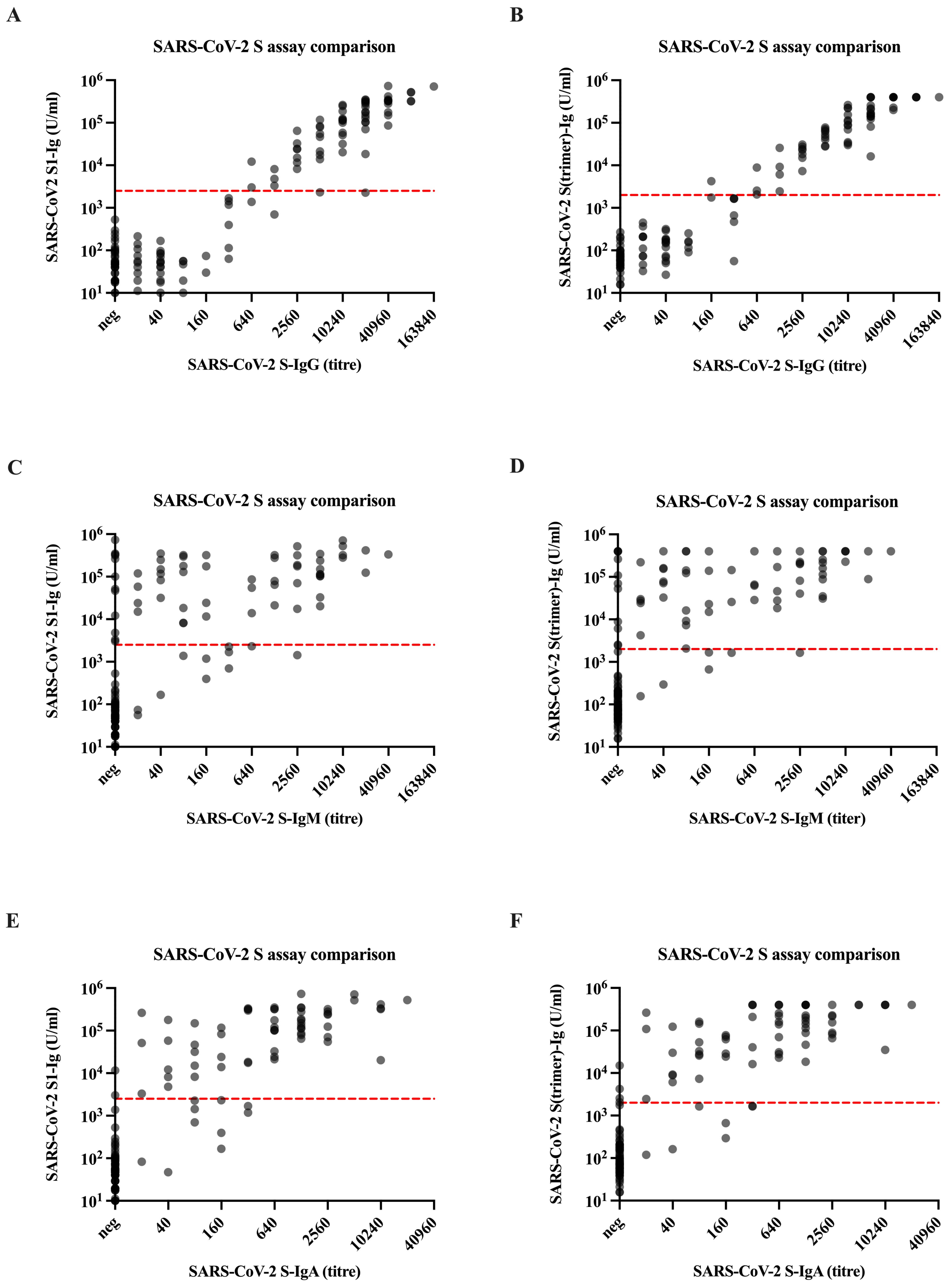

Characterisation of a live CBA for the detection of SARS-CoV-2 S antibodies. Spearman correlation of (**A**) SARS-CoV-2 S-IgG with S1-Ig (R=0.867), (**B**) SARS-CoV-2 S-IgG with S(trimer)-Ig (R=0.937), (**C**) SARS-CoV-2 S-IgM with S1-Ig (R=0.710), (**D**) SARS-CoV-2 S-IgM with S(trimer)-Ig (R=0.726), (**E**) SARS-CoV-2 S-IgA with S1-Ig (R=0.868), (**F**) SARS-CoV-2-S IgM with S(trimer)-Ig (R=0.862). The cut-off values for the SARS-CoV-2 S1-Ig and S(trimer)-Ig assays are indicated by the dashed red lines. Ig = total immunoglobulin, IgA = immunoglobulin A, IgG = immunoglobulin G, IgM = immunoglobulin M, SARS-CoV-2 = severe acute respiratory syndrome coronavirus, S = SARS-CoV-2 spike protein, S1 = S1 subunit SARS-CoV-2 spike protein, S(trimer) = trimer of SARS-CoV-2 spike protein, U/ml = units/ml.

Supplementary Figure 2

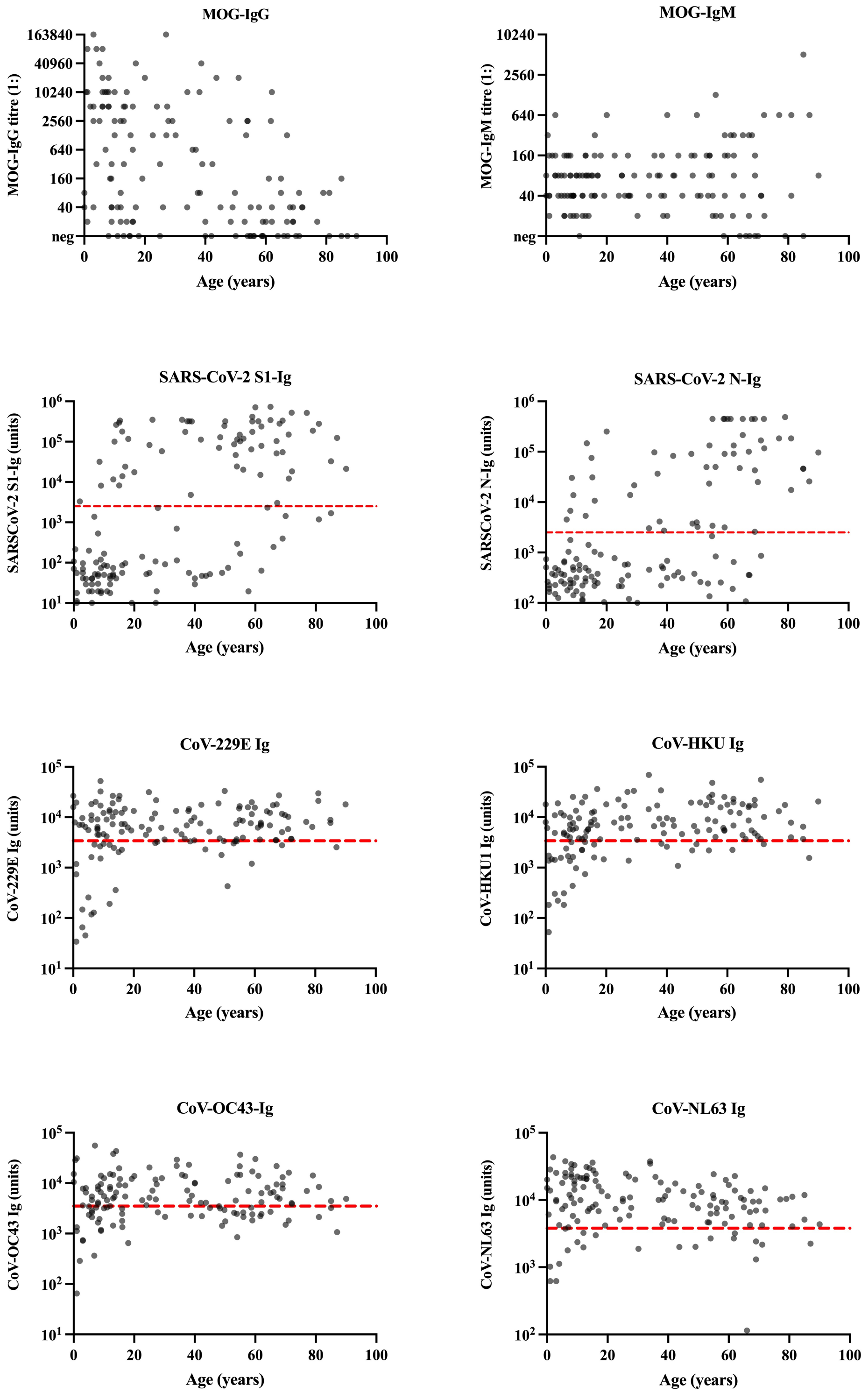

Association of MOG-IgG, MOG-IgM, SARS-CoV-2 S1-Ig, SARS-CoV-2 N-Ig, CoV-229W-Ig, CoV-HKU1-Ig, CoV-OC43-Ig and CoV-NL63-Ig antibodies with age. The cut-off values for the SARS-CoV-2 and CoV-229E, CoV-HKU1, CoV-OC43 and CoV-NL63 antibody assays are indicated by the dashed red lines. CoV = coronavirus, Ig = total immunoglobulin, IgG = immunoglobulin G, IgM = immunoglobulin M, MOG = myelin oligodendrocyte glycoprotein, N = SARS-CoV-2 nucleocapsid protein, SARS-CoV-2 = severe acute respiratory syndrome coronavirus, S = SARS-CoV-2 spike protein, S1 = S1 subunit SARS-CoV-2 spike protein.

Supplementary Figure 3

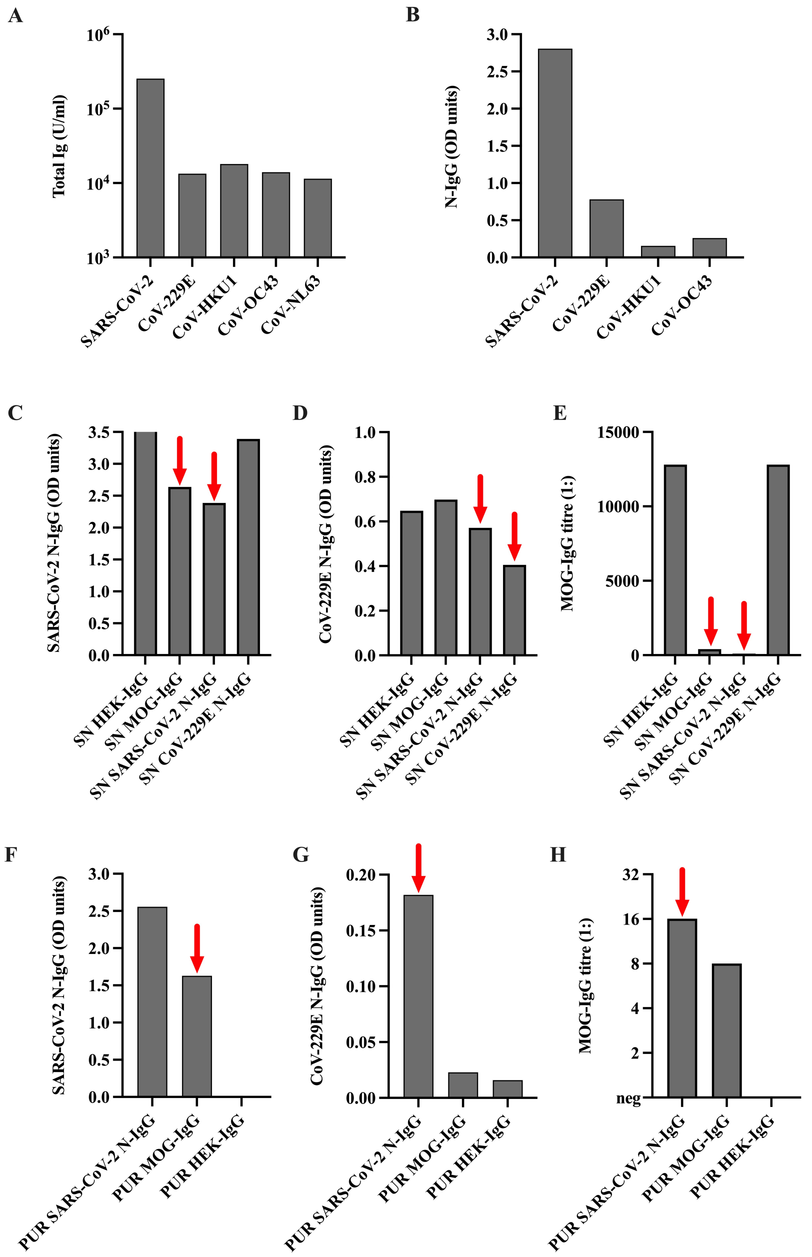

Immunological cross-reactivity between MOG, SARS-CoV-2 N and CoV-229E N proteins of sample #2 (MOGAD after COVID-19). (**A**) Binding of total serum Ig antibodies to SARS-CoV-2 N, CoV-229E, CoV-HKU1, CoV-OC43 and CoV-NL63. (**B**) Binding of serum IgG antibodies to SARS-CoV-2 N, CoV-229E N, CoV-HKU1 N and CoV-OC43 N. (**C-E**) Binding of antigen-specific/control depleted or quenched supernatants to: (**C**) SARS-CoV-2 N (ELISA) (**D**) CoV-229E N (ELISA) and (**E**) MOG (CBA) (**F-G**) Binding of purified human IgG fractions against SARS-CoV-2 N and MOG to (**F**) SARS-CoV-2 N (ELISA) (**G**) CoV-229E N (ELISA) and (**H**) MOG (CBA); arrows indicate observed depletion/quenching of supernatants compared to EGFP and cross-reactive binding of purified antibody fractions. SN HEK-IgG = control immunoglobulin G (IgG) depleted supernatant using HEK293 cells, SN MOG-IgG = myelin oligodendrocyte glycoprotein IgG depleted supernatant using HEK293 expressing human MOG; SN SARS-CoV-2 N-IgG = supernatant quenched with soluble SARS-CoV-2 nucleocapsid protein; SN CoV-229E N-IgG = supernatant quenched with soluble CoV229E nucleocapsid protein.

Supplementary Figure 4

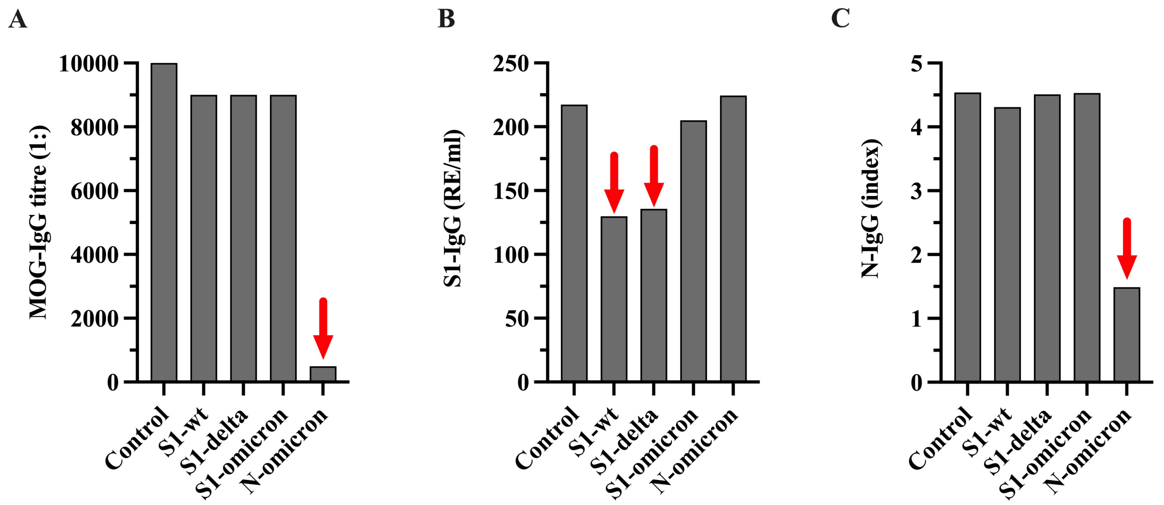

Immunological cross-reactivity between MOG and SARS-CoV-2 S protein variants (recombinant spike protein S1 of wildtype, delta and omicron variants) of sample #2 (MOGAD after COVID-19). (**A**) MOG-IgG titre (CBA), (**B**) SARS-CoV-2 S1 (ELISA) and (**C**) SARS-CoV-2 N (ELISA). Arrows indicate observed depletion/quenching of supernatants compared to control and cross-reactive binding of purified antibody fractions. IgG = immunoglobulin G, MOG = myelin oligodendrocyte glycoprotein, RE/ml = relative units / ml, wt = wildtype.

Supplementary Figure 5

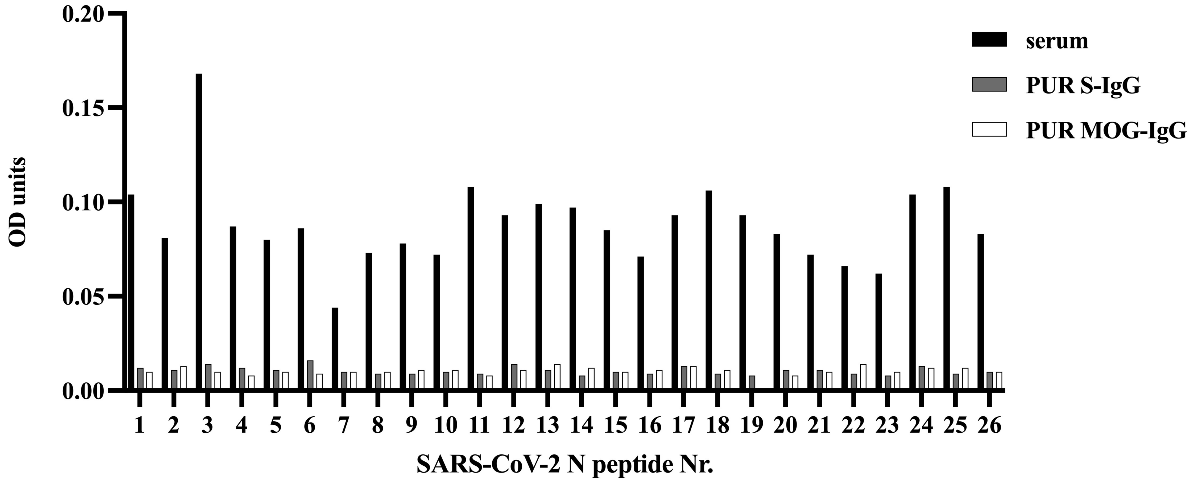

Binding of serum and purified human immunoglobulin G (IgG) fractions (PUR) against SARS-CoV-2 spike protein (S) and myelin oligodendrocyte glycoprotein (MOG) from sample Nr. 2 to 26 overlapping 20mer peptides of SARS-CoV-2 nucleocapsid protein (N) 1-242. No significant binding of IgG antibodies to any specific linear epitope is observed, both in serum and purified antibody fractions (PUR S-IgG acting as control). OD = optical density.

Supplementary Figure 6

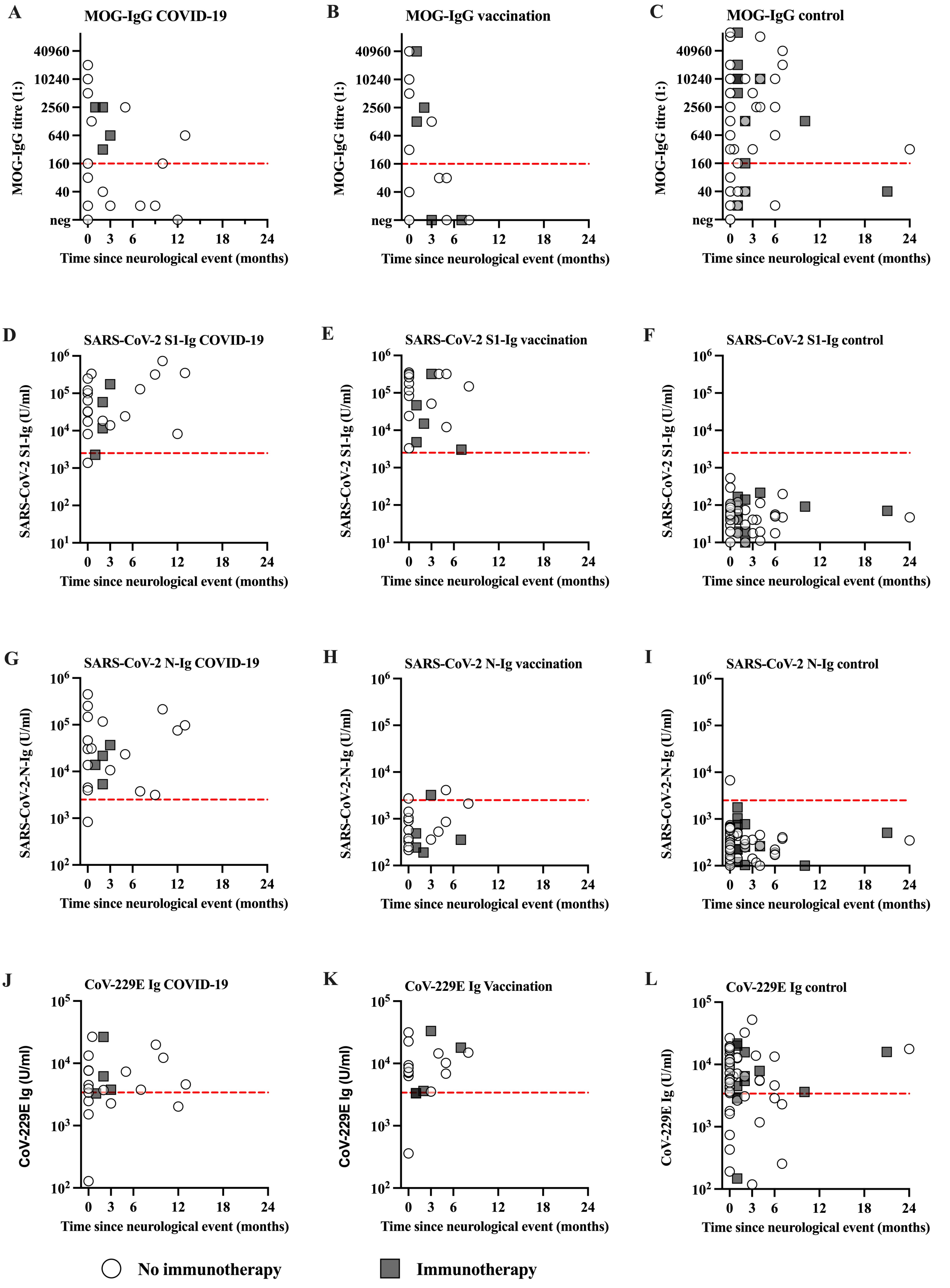

Association of MOG-IgG (A-C), SARS-CoV-2 N-Ig (D-F), SARS-CoV-2 S1-Ig (G-I) and CoV-229 Ig (J-L) antibodies in serum samples from patients after SARS-CoV-2 infection (COVID-19; A, D, G, J), vaccination (B, E, H, K) or controls without SARS-CoV-2 antibodies (C, F, I, L) with time from the neurological event to sampling (in months) and immunosuppressive therapy at sampling. The cut-off values for the MOG-IgG, SARS-CoV-2 N-IgG and S1-Ig and CoV-229E Ig antibody assays are indicated by the dashed red lines.CoV = coronavirus, COVID-19 = coronavirus disease 2019, Ig = total immunoglobulin, IgG = immunoglobulin G, MOG = myelin oligodendrocyte glycoprotein, N = SARS-CoV-2 nucleocapsid protein, SARS-CoV-2 = severe acute respiratory syndrome coronavirus, S1 = S1 subunit SARS-CoV-2 spike protein.
